# Novel oncogene 5MP1 reprograms c-Myc translation initiation to drive malignant phenotypes in colorectal cancer

**DOI:** 10.1016/j.ebiom.2019.05.058

**Published:** 2019-06-04

**Authors:** Kuniaki Sato, Takaaki Masuda, Qingjiang Hu, Taro Tobo, Sarah Gillaspie, Atsushi Niida, Mackenzie Thornton, Yousuke Kuroda, Hidetoshi Eguchi, Takashi Nakagawa, Katsura Asano, Koshi Mimori

**Affiliations:** a Department of Surgery, Kyushu University Beppu Hospital, 4546 Tsurumihara, Beppu, Oita 874-0838, Japan; b Department of Otorhinolaryngology, Graduate School of Medical Sciences, Kyushu University, 3-1-1 Higashi-ku, Fukuoka, Fukuoka 860-8556, Japan; c Department of Clinical Laboratory Medicine and Pathology, Kyushu University Beppu Hospital, 4546 Tsurumihara, Beppu, Oita 874-0838, Japan; d Molecular Cellular and Developmental Biology Program, Division of Biology, Kansas State University, Manhattan, KS 66506, USA; e Division of Health Medical Computational Science, Health Intelligence Center, Institute of Medical Science, University of Tokyo, 4-6-1 Shirokanedai, Minato-ku, Tokyo 108-8639, Japan

**Keywords:** Translational reprogramming, Colorectal cancer, 5MP1, c-Myc, Tumor heterogeneity, Oncogene

## Abstract

**Background:**

Translational reprogramming through controlled initiation from non-AUG start codons is considered a crucial driving force in tumorigenesis and tumor progression. However, its clinical impact and underlying mechanism are not fully understood.

**Methods:**

Using a bioinformatics approach, we identified translation initiation regulator *5MP1/BZW2* on chromosome 7p as a potential oncogenic driver gene in colorectal cancer (CRC), and explored the biological effect of 5MP1 in CRC in vitro or in vivo. Pathway analysis was performed to identify the downstream target of 5MP1, which was verified with transcriptomic and biochemical analyses. Finally, we assessed the clinical significance of *5MP1* expression in CRC patients.

**Findings:**

5MP1 was ubiquitously amplified and overexpressed in CRC. 5MP1 promoted tumor growth and induced cell cycle progression of CRC. c-Myc was identified as its potential downstream effector. *c-Myc* has two in-frame start codons, AUG and CUG (non-AUG) located upstream of the AUG. 5MP1 expression increased the AUG-initiated c-Myc isoform relative to the CUG-initiated isoform. The AUG-initiated c-Myc isoform displayed higher protein stability and a stronger transactivation activity for oncogenic pathways than the CUG-initiated isoform, accounting for *5MP1*-driven cell cycle progression and tumor growth. Clinically, high *5MP1* expression predicts poor survival of CRC patients.

**Interpretation:**

*5MP1* is a novel oncogene that reprograms *c-Myc* translation in CRC. 5MP1 could be a potential therapeutic target to overcome therapeutic resistance conferred by tumor heterogeneity of CRC.

**Fund:**

Japan Society for the Promotion of Science; Priority Issue on Post-K computer; National Institutes of Health; National Science Foundation; KSU Johnson Cancer Center.

**Research in context**

**Evidence before this study**

Recent large-scale proteogenomic studies have revealed a low correlation between mRNA and protein expression levels in human cancer, suggesting that genome-wide coordination of translational control or “translational reprogramming” is a crucial driving force in tumorigenesis or tumor progression. Moreover, several studies have reported that genomic alteration leading to amplification or deletion of translational regulator genes contribute to malignancy. Importantly, our previous genomic studies showed that amplification of chromosome 7p is ubiquitously observed in CRC. These findings motivated us to investigate novel oncogenes that induce translational reprogramming and contribute to malignant progression caused by genomic rearrangement in CRC.

**Added value of this study**

In this study, we showed that a translation initiation regulatory factor *5MP1* on chromosome 7p is frequently amplified in CRC. We provide the first experimental and clinical evidence that 5MP1 acts as an oncogenic driver gene and controls the selection of translational start codons of c-Myc oncogene. Amplified 5MP1 promotes tumor growth and cell cycle progression by promoting the oncogenic function of c-Myc by reprogramming the ratio of CUG to AUG start codon usage and increasing the oncogenic AUG-initiated c-Myc isoform. Clinically, the high 5MP1 expression is associated with metastasis and predicts poor survival of CRC patients.

**Implications of all the available evidence**

5MP1 is a novel oncogene of CRC which contributes to the malignant phenotype of CRC at least in part through the reprograming translation of c-Myc oncogene. Since the amplification of 5MP1 on chromosome 7p is ubiquitously observed in CRC, 5MP1 would serve not only as a prognostic biomarker but also as a promising therapeutic target to overcome therapeutic resistances conferred by tumor heterogeneity.

Alt-text: Unlabelled Box

## 1 Introduction

Translation or mRNA-dependent protein synthesis is a crucial process of gene expression that directly determines the abundance of cellular proteome [1,2]. Attempts to integrate large-scale cancer genomics data such as The Cancer Genome Atlas (TCGA) with the proteomics data strongly suggest that translational regulation can play a significant role in determining phenotypic outcomes of cancer [3,4]. The initiation phase of translation governed by eukaryotic initiation factors (eIFs) is rate-limiting for protein synthesis, and hence considered to be the targets of translational control [5]. Several oncogenes such as c-Myc, mTOR, and RAS affect the activity of these factors and induce selective translation of mRNAs that encode proteins involved in proliferation, angiogenesis and stress responses [6,7]. Genome-wide translational profiling through ribosome protection (ribosome profiling) revealed how these oncogene-induced changes in eIF activities alter the global translational landscape and promote tumor initiation [8,9].

Genetic alterations that contribute to malignancy includes the amplification or deletion of genes encoding eIFs or translational regulators that bind them [[10], [11], [12], [13]]. Pan-cancer genomic database analyses revealed that the arm-level amplification frequency and the oncogene density are positively correlated, suggesting that the amplification of chromosome arms is not only a hallmark but also a driving force during the evolution of cancer [14]. The frequent arm-level amplification sites in cancer are chromosome 7p, 8q and 20q carrying major oncogenes such as EGFR (7p), c-Myc (8q), AURKA (20q) [15,16]. These studies motivated us to investigate novel oncogenes that induce translational reprogramming and contribute to malignant progression caused by genomic rearrangement.

Recently, we showed that the amplification of the short arm of chromosome 7 (chromosome 7p) is ubiquitously observed in *all* regions of an individual colorectal tumor by performing multiregional genomic analysis of colorectal cancer (CRC), one of the most common types of cancer worldwide [17]. Furthermore, we demonstrated that the amplification of chromosome 7p is ubiquitously observed even in the premalignant state of CRC (i.e., colorectal adenoma) by the same approach [18]. These observations suggest that the amplification of chromosome 7p is one of the primary and predominant driver events in the tumorigenesis of CRC, an idea further supported by several reports that the amplification of chromosome 7p is commonly observed not only in CRC but also in adenoma [[19], [20], [21]]. We recently identified the oncogene PSPH on chromosome 7p via our integrated screening approach of CRC datasets [22].

More importantly, we identified the translation initiation regulator eIF5-mimic protein 1 (5MP1) as a novel potential oncogene on chromosome 7p in this screening. 5MP is so-named because of its partial homology with the essential factor eIF5 [23] (not to be confused with eIF5A, the hypusinated elongation factor, or eIF5B involved in ribosomal subunit joining during translation initiation) [24]. Humans encode two copies of 5MP, 5MP1/BZW2 and 5MP2/BZW1 [25]. Accurate translation initiation from AUG codons requires a sophisticated mechanism, allowing the gatekeeper, eIF1, to block mis-initiation from non-AUG codons [26,27]. eIF5 sets the stage for initiation through its GTPase activation protein (GAP) function and yet acts in disfavor of eIF1 anchoring to the initiating ribosome [[28], [29], [30]]. 5MP lacks the GAP function and hence is considered to be a translational regulatory protein [23]. However, 5MP contributes to accurate initiation by competing with eIF5 and shifting the factor interaction in favor of eIF1 anchoring [31]. Thus, 5MP overexpression generally represses non-AUG translation, affecting genome-wide translation profile and hence proteome [31]. Translation from certain non-AUG codons, such as GUG and UUG, is permitted in prokaryotes and utilized for gene regulation [32], and yet has been thought to be rare in eukaryotes. However, recently developed ribosome profiling studies suggested frequent non-AUG translation in the leader region of eukaryotic mRNAs. The role of altered translation initiation in the dysregulation of biological processes leading to human diseases is currently an important subject under extensive investigation [5].

We previously showed by a c-Myc-luciferase reporter assay that 5MP1 expression increases the expression ratio of c-Myc isoform 1 vs 2, whose translation initiates from an AUG or an upstream CUG codon, respectively [31]. Here we test the hypothesis that the amplified 5MP1 causes translational reprogramming through inhibiting non-AUG initiation of the specific oncogene, c-Myc, and, thereby, contributes to the malignant phenotypes of CRC. Our in vitro and in vivo functional experiments and bioinformatics analysis of CRC uncovered that 5MP1 is an oncogene, controlling the translation initiation site of c-Myc. Furthermore, we elucidated the clinical significance of 5MP1 expression in CRC patients. We propose that translational reprogramming through start codon selection plays an important role in cancer development.

## 2 Materials and methods

### 2.1 Cell lines

HCT116, HEK293T and KMST-6 cells were cultured in Dulbecco's-modified Eagle Medium (Life Technologies) supplemented with 10% fetal bovine serum (FBS), penicillin, streptomycin and l-glutamine. SW480 cells were cultured in RPMI 1640 (Life Technologies) supplemented with 10% FBS, penicillin, streptomycin and l-glutamine. LoVo cells were cultured in Ham's F12 medium supplemented with 20% FBS, penicillin, streptomycin and l-glutamine. RCM-1 cells were cultured in 50% RPMI 1640 and 50% Ham's F12 medium supplemented with 10% FBS, penicillin, streptomycin and l-glutamine. All cells were maintained in a humidified atmosphere containing 5% CO_2_ at 37 °C. See also Table S1 for cell lines and sources used in the study.

### 2.2 Murine xenograft model

All animal procedures were performed in compliance with the Guidelines for the Care and Use of Experimental Animals established by the Committee for Animal Experimentation of Kyushu University. Five-week-old female BALB/c nu/nu mice were purchased from Japan SLC, Inc. and maintained under specific pathogen-free conditions. For subcutaneous xenograft assays, 1 × 10^6^ HCT116 cells transfected with 5MP1 expressing vector or empty vector were suspended in 100 μl of serum-free RPMI-1640 medium and subcutaneously injected bilaterally into nude mice. Tumor sizes were measured every three days using a Vernier caliper and calculated using the following formula: tumor volume = length × width^2^ × 0.5. The mice were sacrificed for analysis at 21 days after injection, and the collected tumors were fixed with 10% formalin.

### 2.3 Patients with CRC and collection of clinical samples

All protocols used in this study were approved by the local ethics review board of Kyushu University. A total of 122 patients with CRC who underwent surgical resection of a primary tumor at Kyushu University Beppu Hospital and the affiliated hospitals were enrolled in this study. Clinicopathological factors and clinical stage were classified using the tumor-node-metastasis (TNM) system of classification. All patients were treated in accordance with the Japanese Society of Cancer of the Colon and Rectum Guidelines for the Treatment of Colorectal Cancer. Written informed consent was obtained from all patients. Resected tumor tissues and paired non-neoplastic tissues (NNT) of colorectal mucosa were immediately stored in RNAlater (Thermo Fisher), frozen in liquid nitrogen and kept at −80 °C until RNA extraction. For immunohistochemical staining, the formalin-fixed paraffin-embedded sections of CRC patients were obtained from Kyushu University Beppu Hospital Department of Clinical Laboratory Medicine.

### 2.4 Plasmid construction and generation of stable cell lines

pCDF1-MCS2-EF1-Puro vector was purchased from System Bioscience. Complementary DNA encoding human 5MP1 and c-Myc were subcloned into pCDF1-MCS2-EF1-Puro. For the construction of MYC isoform 2 vector, site-directed mutagenesis was performed using a KOD-Plus-Mutagenesis kit (Toyobo) according to the manufacturer's instructions. All plasmids generated in this study were verified by sequencing. Transfection of cell lines with these plasmids was performed using Lipofectamine 3000 (Thermo Fisher) according to the manufacturer's instructions. After 24 h of transfection, the cells were subjected to selection in medium containing puromycin. Cells stably expressing each recombinant protein were pooled for experiments.

### 2.5 siRNA-mediated knockdown experiments

5MP1-specific siRNAs and negative control siRNA were purchased from Thermo Fisher and Santa Cruz, respectively. Transfection of the CRC cell lines with siRNA oligonucleotides was performed using Lipofectamine RNAiMAX (Thermo Fisher) according to the manufacturer's instructions. Transfected cells were used for downstream analysis.

### 2.6 Total RNA extraction and reverse transcription-quantitative polymerase chain reaction (RT-qPCR)

Total RNA from cell lines and tissues was extracted using ISOGEN-II (Nippon Gene). One μg of RNA was reverse transcribed to cDNA using M-MLV reverse transcriptase according to the manufacturer's instructions (Thermo Fisher). qPCR was performed in triplicate using LightCycler FastStart DNA Master SYBR Green I (Roche) as previously described [33]. The expression levels of genes of interest were normalized by *GAPDH* or *RPS18* mRNA as an internal control, and they are expressed as values relative to the expression level of cDNA from qPCR Human Reference Total RNA (Clontech).

### 2.7 Protein extraction and immunoblotting

For total protein extraction, cells were lysed in lysis buffer (25 mM Tris-HCl at pH 7.5, 150 mM NaCl, 0.2 mM EDTA, 0.1% NP40 and 5% glycerol) containing protease inhibitor cocktail (Bio Vision) and PhosStop phosphatase inhibitor (Roche) on ice. The cell lysates were sonicated for 5 min and centrifuged at 14,000 rpm for 10 min at 4 °C. The supernatants were collected and protein concentrations were determined using the BCA Protein Assay Kit (Thermo Fisher). Equal amounts of lysate were boiled at 98 °C for 5 min with SDS sample buffer. Proteins were electrophoresed via sodium dodecyl sulfate-polyacrylamide gel electrophoresis (SDS-PAGE) in 4–20% or 10% Novex wedge-well Tris-glycine polyacrylamide gels (Life Technologies) and transferred to Immobilon-P PVDF membranes (Millipore) at 70 V for 4 h at room temperature or 30 V overnight at 4 °C. Nonspecific binding sites were blocked with blocking buffer (Tris-buffered saline and 0.1% Tween-20 with 5% nonfat milk) for 1 h at room temperature, and the blot was incubated with specific primary antibodies in blocking buffer (anti-5MP1 at 1:500 dilution, anti-eIF5 at 1:500 dilution, anti-c-Myc at 1:10000 dilution, anti-CDK2 at 1:1000 dilution, Cell Cycle WB Cocktail at 1:250 dilution, anti-β-actin at 1:1000 dilution) at 4 °C overnight. After washing, the blots were incubated with the appropriate secondary antibody conjugated with horseradish peroxidase for 1 h at room temperature. The blots were washed again and detected using a FUSION SOLO S (VILBER). See also Table S1 for more information regarding antibodies used in the assays.

### 2.8 Immunohistochemical analysis

Immunohistochemical staining of CRC cases and mouse xenograft tumors was performed on formalin-fixed, 4-μm-paraffin-embedded sections. The sections were deparaffinized and treated with citrate buffer for epitome retrieval, and endogenous peroxidases were blocked in 3% H_2_O_2_. The primary antibodies used were as follows: anti-5MP1 antibody (GeneTex; 1:500), anti-c-Myc antibody (Y69; Abcam; 1:500), anti-eIF5 antibody (GeneTex; 1:1000), and anti-Ki67 antibody (Abcam; 1:10000). REAL Envision™ kit (Dako) was used for detection. The sections were counterstained with hematoxylin. The immunohistochemistry scoring was performed using IHC Profiler [34], a plugin package of ImageJ software. Immunohistochemical staining intensity of colorectal cancer tissues was scored as low, medium or high for 5MP1 and eIF5, and as low and high for c-Myc. Tumor histology was independently reviewed by an experienced research pathologist in Kyushu University Beppu Hospital Department of Clinical Laboratory Medicine. See also Table S1 for more information regarding the antibodies in the assays.

### 2.9 Cell proliferation assay

Cell proliferation was evaluated by performing MTT assays using the Cell Proliferation Kit 1 (Roche) according to the manufacturer's protocol. In brief, cells were seeded at 3000 cells per well in triplicate onto 96-well plates in 100 μl of medium. The colour reaction was quantitated using an iMark microplate reader (Bio-Rad) at 570 nm with a reference filter of 650 nm.

### 2.10 Colony formation assay

For overexpression studies, cells were plated at a density of 3000 cells/well in triplicates onto 6-well plates and incubated at 37 °C under 5% CO_2_ with antibiotic selection. For siRNA-mediated 5MP1 knockdown studies, the cells were plated at a density of 3000 cells/well onto 6-well plates and incubated at 37 °C under 5% CO_2_ overnight. Cells were then transfected with si5MP1 or negative control siRNA using Lipofectamine RNAiMAX (Thermo Fisher) in triplicates. After 10 days, the colonies were stained using a Differential Quick Stain Kit (Sysmex) according to the manufacturer's instructions. Visible colonies were photographed using a FUSION SOLO S. Colony counts were determined using the ImageJ software.

### 2.11 Sphere formation assay

Cells were seeded onto 6-well ultralow attachment plates (Corning) at a density of 1000 cells/well in a mix of 50% DMEM and 50% Ham's F12 containing 20 ng/ml EGF (PeproTech), 20 ng/ml bFGF (PeproTech) and B27 Supplement (Invitrogen). Triplicate wells were prepared for each cell. After 2 weeks, visible spheres were manually counted using a microscope. Each independent experiment was performed three times.

### 2.12 Flow cytometry and cell cycle analysis

Cells were synchronized at the G1 phase of the cell cycle via serum starvation for 72 h and restimulated by changing medium containing 10% FBS. The cells were harvested, washed with PBS twice, and fixed in 70% ethanol at −20 °C. The fixed cells were incubated in 0.25 mg/ml RNase for 30 min at 37 °C and washed with PBS. Subsequently, the cells were incubated in 5 mg/ml propidium iodide (Sigma Aldrich) for 30 min at room temperature in the dark. Cell cycle acquisition was performed on a SH800 cell sorter (Sony Biotechnology). For siRNA-mediated 5MP1-knockdown cells, siRNAs were transfected to cells after 48 h of serum starvation. Cells were restimulated by changing medium containing 10% FBS after 24 h of transfection and then harvested after 12 h of re-stimulation. Cell cycle acquisition was performed in the same manner.

### 2.13 Apoptosis assay

After 48 h of transfection of 5MP1-specific siRNAs or negative control siRNA, apoptosis of the CRC cells treated with the siRNAs was measured using the Annexin V-FITC Apoptosis Detection Kit (Abcam) according to manufacturer's instructions. Cells were subsequently analyzed using a SH800 cell sorter. For 5MP1-overexpressed HCT116 cells, apoptosis of the cells was measured in the same manner after 48 h of treatment with 5-fluorouracil (5-FU, Wako) at a final concentration of 0.1–100 μM.

### 2.14 High-throughput inhibitor screening assay

A SCADS inhibitor kit was provided by the Screening Committee of Anticancer Drugs (http://scads.jfcr.or.jp/kit/kit.html). This kit contains 374 chemical compounds and anticancer agents targeting known oncogenic kinases, metabolic and signaling pathways. 5MP1-overexpressed HCT116 cells and control cells were seeded in triplicate onto 96-well plates at a density of 12,500 cells/well. Twenty-four hours after plating, cells were treated with chemicals dissolved in dimethyl sulfoxide (DMSO) at a final concentration of 1 μM. DMSO-treated wells were used in each plate as a negative control. Ninety-six hours after treatment, cell viability was assessed by a MTT assay.

### 2.15 Cycloheximide Chase assay

Cells were treated with 50 mM cycloheximide (Abcam) and harvested at the indicated time points. Total protein was extracted from the cells and subjected to Western blot analysis. The half-lives of the proteins of interest were calculated as previously described [35].

### 2.16 Luciferase assay

Luciferase assay of HEK293T cells was performed using Dual-Glo® Luciferase Assay System (Promega) as previously described [31]. The WT c-Myc_408 plasmid is a Firefly luciferase reporter plasmid with the 408-base *MYC* mRNA leader covering its CUG and AUG start codons. These start codons are in-frame to the luciferase-reading frame. The mutant plasmid used here is a derivative of the WT plasmid with its CUG start codon altered to CUC.

### 2.17 TCGA data analysis and selection of candidate genes

We obtained RNA-seq data of 623 CRC patients, DNA copy number data of 615 CRC patients and clinical data in The Cancer Genome Atlas (TCGA) from the Broad Institute's Firehose (http://gdac.broadinstitute.org/runs/stddata__2016_01_28/data/COADREAD/20160128/) and NIH Genomic Data Commons (GDC) Legacy Archive (https://portal.gdc.cancer.gov/legacy-archive). The RNA-seq data and the DNA copy number data also included the expression profiles of 51 and 107 paired NNT samples, respectively. We integrated the copy number data with the RNA-seq data along with clinical data using R version 3.2.3 (The R Foundation for Statistical Computing, Vienna, Austria) and R studio version 0.99a. The candidate genes were extracted from a total of 426 genes on chromosome 7p that satisfied the following two criteria: 1) the gene in question was significantly overexpressed in tumor tissues compared to NNT (>1.5-fold change, Mann-Whitney *U* test *p*-value <0.01) and 2) the DNA copy number and mRNA expression levels had to be positively correlated with each other. The threshold of the correlation coefficient was determined as 0.4. For analyzing TCGA liquid chromatography-tandem mass spectrometry (LC-MS/MS) data, a normalized dataset of 90 CRC samples and 30 NNT samples were obtained from the previous report [3], and expression levels of proteins of interest were analyzed. Overrepresentation Enrichment Analysis (ORA) of the LC-MS/MS dataset was performed using the LinkedOmics Website (http://www.linkedomics.org/) [36].

### 2.18 Estimation of dN/dS ratios in TCGA CRC dataset

We obtained Mutation Annotation Format (MAF) files of 580 CRC patients in TCGA from NIH GDC Legacy Archive. The somatic mutation data of each patient was extracted from these MAF files using R. The estimation of dN/dS ratios for missense mutations of each gene was performed using R package dNdScv v0.0.1.0 (https://github.com/im3sanger/dndscv), a maximum-likelihood method for estimating dN/dS ratios [37] with default parameters. In this analysis, 19 patients and 771 mutations were excluded for exceeding the limit of mutations per sample and the limit of mutations per gene per sample, respectively. For Fig. S2, 2784 genes without any mutations were excluded to plot the histogram.

### 2.19 RNA sequencing and data analysis

RNA extracted from cell lines were sequenced in Illumina HiSeq 4000 at Beijing Genomics Institutions (Shenzhen, China), and the sequenced data were analyzed in an in-house pipeline Genomon2 v2.5.0 (https://genomon-project.github.io/GenomonPagesR) using the supercomputing system Shirokane 3 (University of Tokyo, Japan). Briefly, sequencing reads were aligned to the human reference GRCh38/hg38 genome by STAR v2.5.2a [38] using Gencode v27 annotations. Gene count tables were generated with htseq-count, a part of the HTSeq framework v0.6.0 [39]. Downstream analyses were carried out using R v3.2.3 and BioConductor v3.6 [40]. Normalizing the count data and detection of differentially expressed genes was carried out with DESeq2 v1.10.1 [41]. For sample clustering and principal component analysis, genes with zero counts across all samples were removed from the analysis. For differential expression analysis, the likelihood ratio test was used to extract significant differences across all three conditions. Gene Ontology (GO) analysis was performed using the DAVID tool version 6.8 Beta [42]. Gene set enrichment analysis (GSEA) [43] was performed using GSEA MSigDB v5.0 (Broad Institute, http://www.broadinstitute.org/gsea/msigdb/index.jsp).

### 2.20 Statistical analysis

**Fig. 1:**
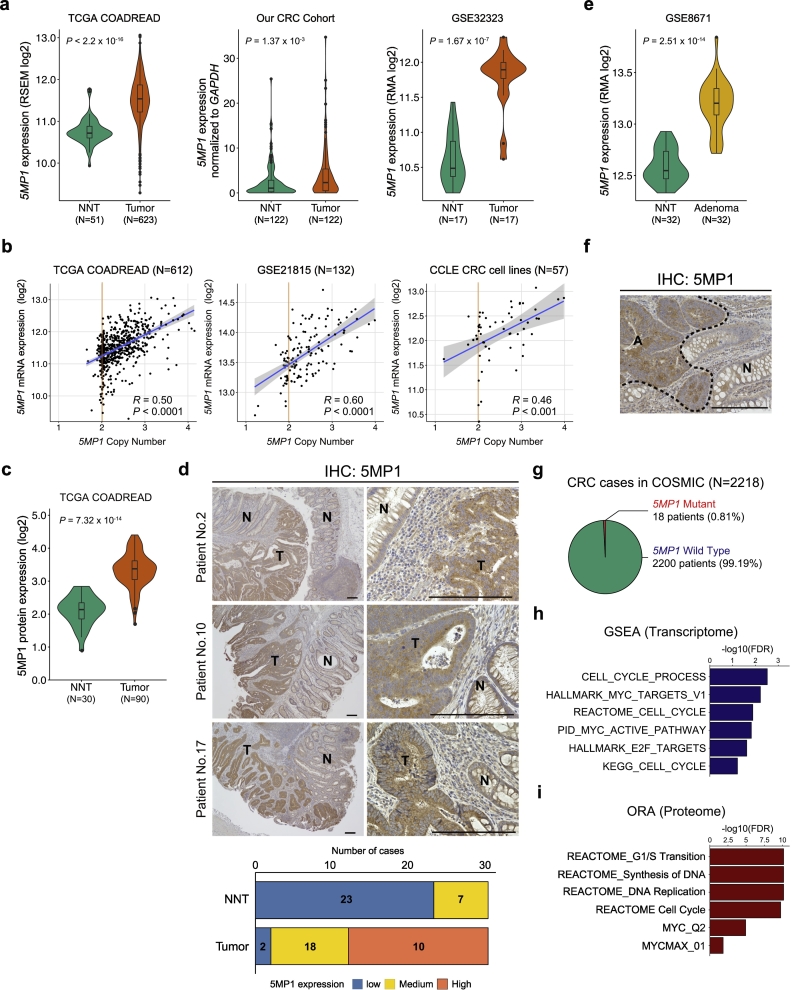
A translation initiation regulatory factor 5MP1 on chromosome 7p is amplified in CRC. (a) Violin plots of *5MP1* mRNA expression levels in CRC tissues and non-neoplastic tissues (NNT) of colorectal mucosa in the TCGA COADREAD dataset (left), our dataset obtained by qRT-PCR (medium), and GSE32323 (right). P represents *p*-values from two-sided Mann-Whitney *U* tests. (b) Correlation between the *5MP1* copy number and the *5MP1* mRNA expression levels in the TCGA COADREAD dataset (left), GSE21815 (medium), and CRC cell lines in Cancer Cell Line Encyclopedia (CCLE) (right). R represents the Pearson correlation coefficient. (c) Violin plots of 5MP1 protein expression levels in CRC tissues and NNT in the TCGA proteome dataset. P represents p-value from the two-sided Mann-Whitney U test. (d) Representative images of immunohistochemical staining for 5MP1 in CRC tissues (upper). Proportions of 5MP1 levels in tumor tissues and NNT are shown using a three-stage staining score (lower). T, Tumor; N, NNT; Scale bars, 200 μm. (e) Violin plots of *5MP1* mRNA expression levels in colorectal adenoma tissues and NNT in GSE8671. P represents p-value from the two-sided Mann-Whitney U test. (f) Representative image of immunohistochemical staining for 5MP1 in a surgically resected colorectal adenoma tissue. A, Adenoma; N, NNT; Scale bars, 200 μm. (g) Pie chart of *5MP1* mutation distribution in CRC cases of the COSMIC database (*N* = 2218). (h,i) Gene set enrichment analysis (GSEA) of the TCGA COADREAD transcriptome dataset (*N* = 623) (h) and Overrepresentation enrichment analysis of the TCGA COADREAD proteome dataset (*N* = 90) (i) showing six significantly enriched gene sets involved in cell cycle progression and c-Myc targets that are positively correlated with 5MP1 expression levels. Threshold of False Discovery Rate (FDR) is <0.25 and < 0.05, respectively.

Statistical analyses were performed using JMP Pro v13.0.0 software (SAS Institute) and R version 3.2.3. For continuous variables, statistical analyses were performed using Student's *t*-test. For Fig. 1, associations between the variables were tested by the Mann-Whitney *U* test. Categorical variables were compared using Fisher's exact test. For survival analysis, cases were divided into two groups based on 5MP1 expression levels using the minimum *P*-value approach, which is a comprehensive method to identify the optimal risk separation cutoff point in continuous gene expression measurements for survival analysis in multiple datasets [44]. Overall survival (OS) curves were plotted according to the Kaplan-Meier method and compared using the log-rank test. Univariate and multivariate analyses were performed using the Cox proportional hazards model to identify independent variables predictive of OS. The degree of linearity was estimated by Pearson's correlation coefficient. The differences were considered significant when the *p*-value was lower than 0.05. All data analysis is performed with at least 3 replicates and presented as the mean ± SD of replicates.

### 2.21 Data resources

The RNA-Seq datasets (accession no. GSE118105 and GSE118113) have been deposited to the NCBI Gene Expression Omnibus: https://www.ncbi.nlm.nih.gov/geo/.

### 2.22 Software

Software programs used in this study were obtained from publicly available resources. See also Table S1 for more details.

## 3 Results

### 3.1 A translation initiation regulatory factor 5MP1 on chromosome 7p is amplified in CRC

The amplification of chromosome 7p is frequently observed in CRC (Fig. S1a; also see the amplifiaton of 7q, 8q, 13 or 20). Furthermore, this event is spatiotemporally shared in CRC development as we have reported [17,18]. Thus, we attempted to identify novel potential driver genes that are located on chromosome 7p. For this purpose, we used TCGA datasets including mRNA expression and DNA copy number profiles of 612 CRC patients, as described before (Fig. S1a and b) [22]. From a total of 426 genes located on chromosome 7p, we extracted 20 candidate genes that satisfied the two criteria: 1) overexpression in tumor tissues compared to non-neoplastic tissues (NNT) of colorectal mucosa (> 1.5-fold change, Mann-Whitney *U* test *p* < 0.01) and 2) positive correlation between DNA copy number and mRNA expression levels (Pearson correlation >0.4, p < 0.01). These candidates included several genes that had been reported as oncogenes in CRC (i.e., *NFE2L3*, *MACC1*, and *PSPH*) [[45], [46], [47]]. Among these 20 genes, eIF5-mimic protein 1 (*5MP1*), also known as basic leucine zipper and W2 domains 2 (*BZW2*), was located on the residue 7p21.1. *5MP1* is a translational regulatory gene [23,31,48] and is essential for animal development [25]. 5MP1 regulates the selection of the translation start codon [31] by competing with the translation initiation factor eIF5 for binding to the Met-tRNA-binding factor eIF2 and the ribosome-binding factor eIF3 [23]. In agreement with this report, proteomics analysis demonstrated the tight association of 5MP1 with eIF2 and eIF3 in human cells [48]. Interestingly, 5MP1 has been reported as a potential cancer driver gene by a computational method called the TUSON explorer, which predicts tumor suppressor genes and oncogenes in pan-cancer genomic databases [14].

We observed that *5MP1* mRNA expression levels were elevated in CRC tissues in TCGA, Affymetrix-based CRC tissue dataset GSE32323 [49], and our dataset from reverse transcription-quantitative polymerase chain reaction (RT-qPCR) experiments (Mann-Whitney *U* test, *p* < 2.2 × 10^−16^, *p* = 1.67 × 10^−7^, and *p* = 1.37 × 10^−3^, respectively) (Fig. 1a). In TCGA dataset, only a small population showed the low expression levels of *5MP1*, indicating that *5MP1* downregulation is rarely observed in CRC patients (2.1% of total cases when *5MP1*-low is defined as the ratio of tumor/median of NNT < 0.66, data not shown). However, for the majority of the cases (64.9% of the total cases), *5MP1* was amplified (*5MP1*-amplification was defined here as the log2 copy number ratios of *5MP1* in tumor tissues >0.1 [50]) (Fig. S1c), and its DNA copy number and mRNA expression levels were positively correlated in GSE21815 [51], the CRC cell line detaset (Cancer Cell Line Encyclopedia), and TCGA (Pearson correlation *R* = 0.60, *p* < 0.0001; *R* = 0.46, *p* < 0.001; and *R* = 0.51, p < 0.0001, respectively) (Fig. 1b). The liquid chromatography-tandem mass spectrometry (LC-MS/MS) dataset of CRC patients from TCGA [3] showed the upregulation of 5MP1 protein in tumor tissues (Fig. 1c). Since a previous study reported that the expression levels of eIFs are different depending on primary sites and tumor grades in CRC [52], we also analyzed the expression levels of 5MP1, dividing the patients into four groups by primary sites and tumor grades. Notably, *5MP1* was significantly overexpressed in tumor tissues compared with NNT in any group, suggesting that overexpression of *5MP1* is a common alteration in CRC (Fig. S1d).

In immunohistochemical analysis, 5MP1 was mainly stained in tumor cells and 60% of the CRC cases showed a positive staining of 5MP1 in tumor lesions, in contrast to weak staining in NNT (Fig. 1d). Interestingly, 5MP1 was overexpressed in colorectal adenoma, a precancerous lesion of CRC (Fig. 1e and f) [53]. While these data might suggest a genetic alteration in 5MP1, the somatic mutations of *5MP1* are rarely observed in CRC; only 0.81% of patients with CRC have the mutations in *5MP1* in the Catalogue of Somatic Mutations in Cancer (COSMIC) (*N* = 2218, Fig. 1g). Moreover, *5MP1* has low dN/dS ratio of 0.39 in TCGA CRC dataset (*N* = 561, Fig. S2). Negative selection in cancer genomes is much weaker than anticipated, and the vast majority of genes accumulate point mutations near neutrally, with dN/dS~1 [37]. Only 850 genes (4.2% of analyzed genes) accumulate 2-fold or less fewer missense (nonsynonymous) mutations than synonymous mutations with dN/dS < 0.5 (Fig. S2). These observations suggest that the wild-type sequence of *5MP1* is important in CRC progression, and that this gene is under purifying selection in CRC evolution. We, therefore, conclude that the amplification of 5MP1, in its wild-type form, is the major and primary alteration in CRC development.

### 3.2 Pathway analysis of 5MP1 in CRC suggests its link to c-Myc

To explore oncogenic pathways that are positively correlated with 5MP1 expression in CRC, we performed Gene Set Enrichment Analysis (GSEA) and Overrepresentation Enrichment Analysis (ORA) using the TCGA RNA-seq dataset and LC-MS/MS dataset, respectively. Notably, the 5MP1 expression is positively and strongly correlated with the expression of gene sets involved in cell cycle progression both in GSEA and ORA (Fig. 1h, i and S1d). Furthermore, the 5MP1 expression is also positively and strongly correlated with the expression of transcriptional target gene sets for c-Myc, which is one of the most frequently activated oncogenes in human cancer [54].

### 3.3 5MP1 promotes the tumor growth of CRC in vitro and in vivo

**Fig. 2:**
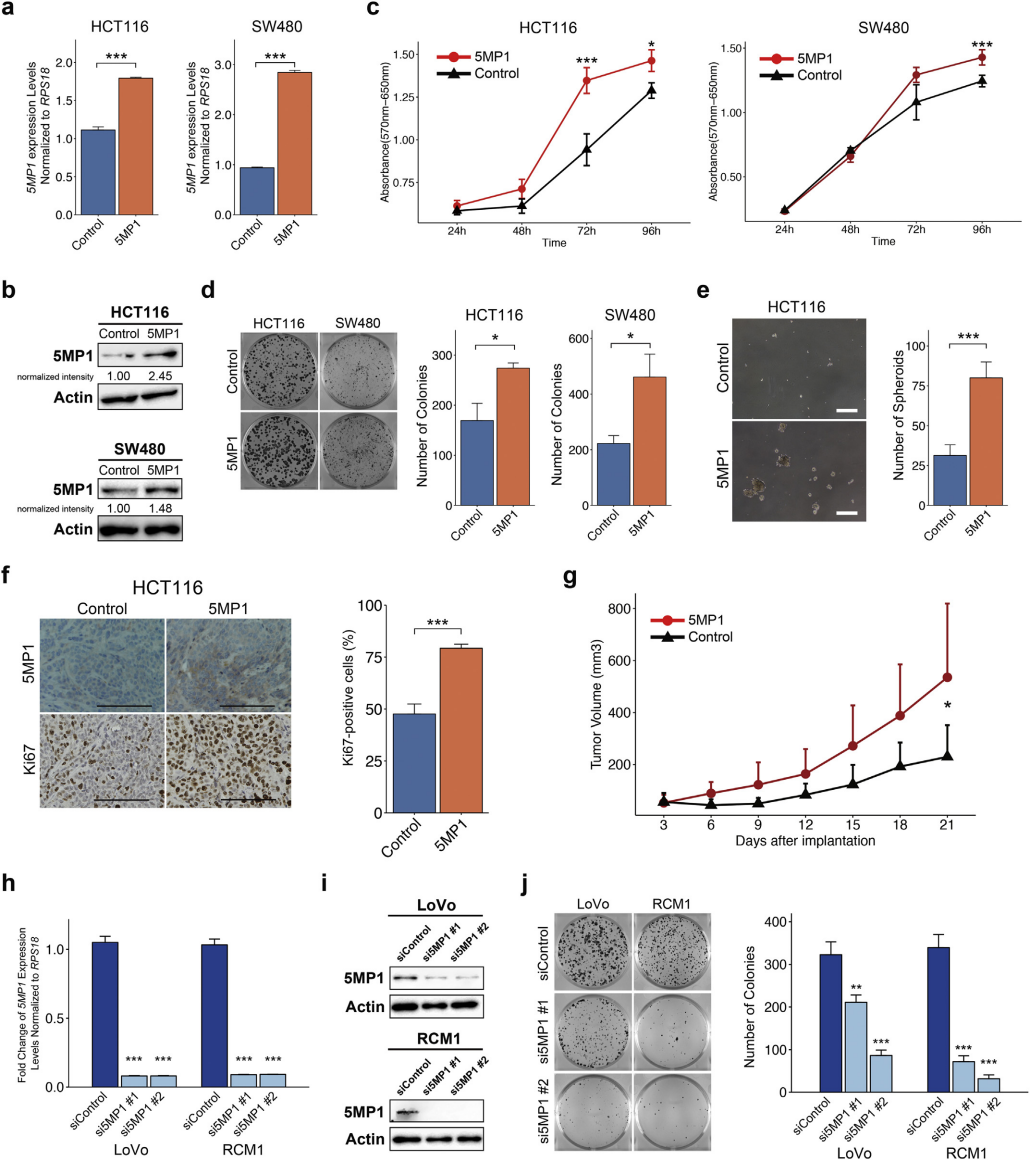
5MP1 promotes tumor growth of CRC. (a) Relative *5MP1* mRNA expression levels, as measured by qRT-PCR, in control cells and 5MP1-overexpressed cells, normalized to *RPS18* mRNA expression (*n* = 3). (b) Western blot of 5MP1 in control cells and 5MP1-overexpressed cells. Actin was used as the loading control for relative protein quantification. The normalized intensities of 5MP1 protein are shown. (c) Proliferation curve of 5MP1-overexpressed cell lines and control cell lines measured by MTT assays (n = 3). (d) Representative images (left) and quantification of colonies (right) of 5MP1-overexpressed cells and control cells at day 14 (n = 3). (e) Representative images (left) and quantification of oncospheres (right) of HCT116 at day 14. Scale bars, 500 μm. (f) Representative images of immunohistochemical staining for 5MP1 and Ki67 in xenografted tumor tissues (left). Bar graphs represent the percentage of Ki67-positive cells in 5MP1-overexpressed tumor tissues and control tumor tissues (right, *n* = 6). Scale bars, 100 μm. (g) The growth curve of xenograft tumors expressing 5MP1 (n = 6) and control (n = 6). (h) Fold change in *5MP1* mRNA expression levels measured by qRT-PCR in the cells transfected with the indicated siRNAs, normalized to RPS18 mRNA expression (n = 3). (i) Western blot of 5MP1 in the cells transfected with the indicated siRNAs. Actin was used as the loading control. (j) Representative images (left) and quantification of colonies (right) of the cells transfected with the indicated siRNAs at day 14 (n = 3). Data represent the mean ± SD. n.s., not significant; (*) *p* < 0.05; (**) *p* < 0.01; (***) *p* < 0.001.

The results of GSEA and ORA motivated us to investigate whether 5MP1 regulates c-Myc-induced cell cycle progression and tumor proliferation. Provided that 5MP1 expression levels vary between different isolates of CRC (Fig. 1), we first measured *5MP1* mRNA expression level in a set of immortalized CRC cell lines (Fig. S3a). We reasoned that CRC cell lines with lower *5MP1* expression would be limited for 5MP1 and hence suited for its overexpression experiments. Thus, we chose two *5MP1*-limited CRC cell lines, HCT116 and SW480, and generated their stably *5MP1*-transfected derivatives (Fig. 2a, b and S3a). The ectopic 5MP1 expression significantly promoted proliferation, colony formation and sphere formation of these CRC cell lines compared to the controls in vitro (Fig. 2c-e, and Fig. S3b and c). Additionally, 5MP1 expression significantly increased the volumes of CRC tumors in xenograft mice models (Fig. 2f and g). Immunohistochemical staining of Ki67 revealed a significant enhancement of proliferation in *5MP1*-transfected tumors (Fig. 2f).

**Fig. 3:**
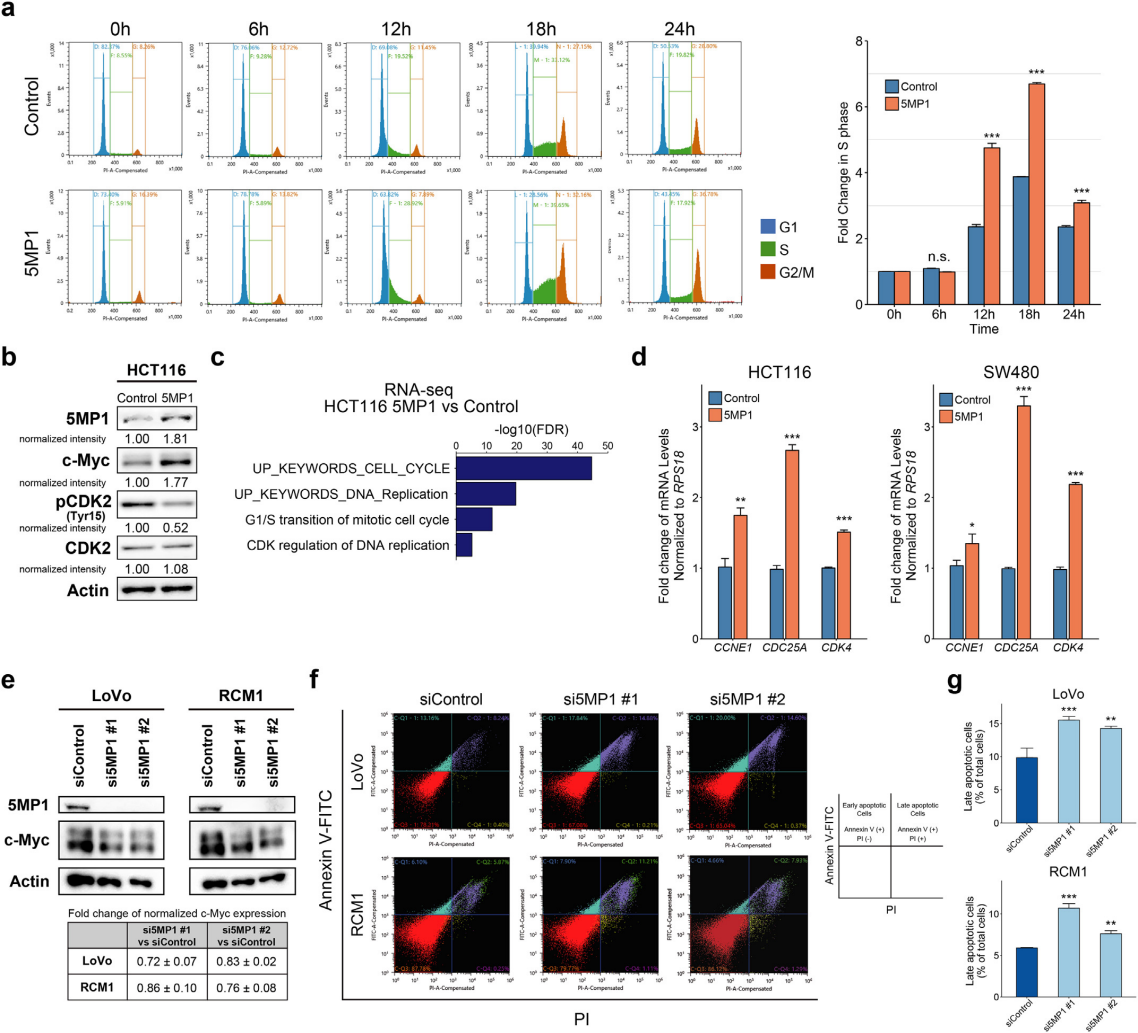
5MP1 promotes cell cycle progression. (a) Cell cycle assay of HCT116 control cells and 5MP1-overexpressed HCT116 cells. Propidium iodide (PI) staining was performed after refeeding of FBS for the indicated time periods (left). Bar graphs represent the fold change in the proportion of S-phase distribution (right). (b) Western blot of 5MP1 and cell-cycle-related proteins in control and 5MP1-overexpressed cells. Actin was used as the loading control for relative protein quantification. The normalized intensities of each protein are shown. (c) Gene Ontology analysis of upregulated genes in 5MP1-overexpressed cells (fold change >1.5) conducted by DAVID (FDR < 0.01). (d) Fold change in mRNA expression levels of cell-cycle-related genes measured by qRT-PCR in control cells and 5MP1-overexpressed cells, normalized to *RPS18* mRNA expression (n = 3). (e) Western blot of 5MP1 and c-Myc in the cells transfected with 5MP1 siRNA (upper). The fold changes of normalized c-Myc protein expression levels in the 5MP1-knockdown cells compared to control cells are shown (lower). Actin was used as the loading control for relative protein quantification. Data represent the mean ± SD of two independent experiments. (f) The representative images of apoptosis rates measured by flow cytometry in LoVo (upper) and RCM1 (lower) CRC cell lines transfected with the indicated siRNAs. (g) The quantification of cell distribution in late apoptosis (Annexin V-FITC^+^/propidium iodide [PI]^+^). Data represent the mean ± SD of three independent experiments. n.s., not significant; (*) p < 0.05; (**) p < 0.01; (***) p < 0.001.

Next, we investigated the effect of 5MP1 knockdown in the *5MP1*-replete LoVo and RCM1 CRC cell lines (Fig. S3a) with two small interfering RNAs (siRNAs) targeting *5MP1*. Both siRNAs targeting *5MP1* strongly reduced 5MP1 expression (Fig. 2h and i; also see Fig. 3e). We also evaluated the expression levels of eIF5 in these cell lines; being a competitor of 5MP1 [31], alteration in its expression may perturb translational control by altered 5MP1 levels. However, we did not observe the changes in eIF5 protein expression levels as expected (Fig. S3d and e). Importantly, knockdown of 5MP1 significantly decreased the number of colonies, suggesting a reduced ability of anchorage-dependent growth (Fig. 2j). These results indicate that 5MP1 positively regulates tumor growth of CRC in vivo and in vitro.

### 3.4 5MP1 promotes the cell cycle progression of CRC through inducing c-Myc

Next, we performed the cell cycle analysis of the *5MP1*-transfected HCT116 derivatives. We observed that *5MP1*-transfected HCT116 cells had significantly higher proportion of cells in S-phase than its control HCT116 cells at 12 h after re-stimulation (Fig. 3a, mean ± SD; 28.18 ± 0.82% and 20.15 ± 0.62% of total counted cells, respectively; *n* = 3; paired *t*-test, *p* < 0.001), suggesting that 5MP1 promotes the G1/S transition of the cell cycle. Also, 5MP1 knockdown partially reduced the proportion of cells in S-phase (Fig. S4a). Importantly, we observed the overexpression of the c-Myc protein and a decrease in Tyr15-phosphorylated CDK2 by Western blotting (Fig. 3b). The dephosphorylation of CDK2 Tyr-15 required for its activation is catalyzed by CDC25A, a transcriptional target of c-Myc [55]. Consistent with these data, Gene Ontology (GO) analysis and GSEA of RNA-seq data derived from *5MP1*-transfected HCT116 cells and its control HCT116 cells showed that gene sets involved in cell cycle progression and G1/S transition of the cell cycle are significantly enriched in the *5MP1*-transfected cells (Fig. 3c and S4b). Furthermore, the qRT-PCR analysis demonstrated that several c-Myc target genes that are involved in cell cycle progressions e.g. *CCNE1*, *CDC25A*, and *CDK4,* are significantly upregulated (Fig. 3d) [56,57]. To reinforce these data, we performed high-throughput inhibitor screening assays using a Screening Committee of Anticancer Drugs (SCADS) inhibitor kit that included 374 chemical compounds (http://scads.jfcr.or.jp/kit/kit.html). This screening revealed that several inhibitors targeting CDK1/CyclinB, CDK2/CyclinE and Aurora kinases, the downstream targets of c-Myc involved in cell cycle progression [57,58], significantly inhibited the growth of the 5MP1-transfected CRC cells (Fig. S4c and d). Thus, these data suggest that 5MP1 accelerates cell cycle progression through inducing c-Myc protein expression.

We did not observe a significant difference in apoptosis between 5MP1-transfected cells and control cells (Fig. S4e). By contrast, siRNA-mediated knockdown of 5MP1 decreased c-Myc protein expression levels and induced apoptosis, which is likely explained by the downregulation of c-Myc as previously described (Fig. 3e-g) [[59], [60], [61]]. These results suggest that 5MP1 expression induces c-Myc expression in CRC.

### 3.5 5MP1 promotes the accumulation of the AUG-initiated c-Myc isoform (isoform 1) by repressing the CUG-initiated c-Myc isoform (isoform 2)

**Fig. 4:**
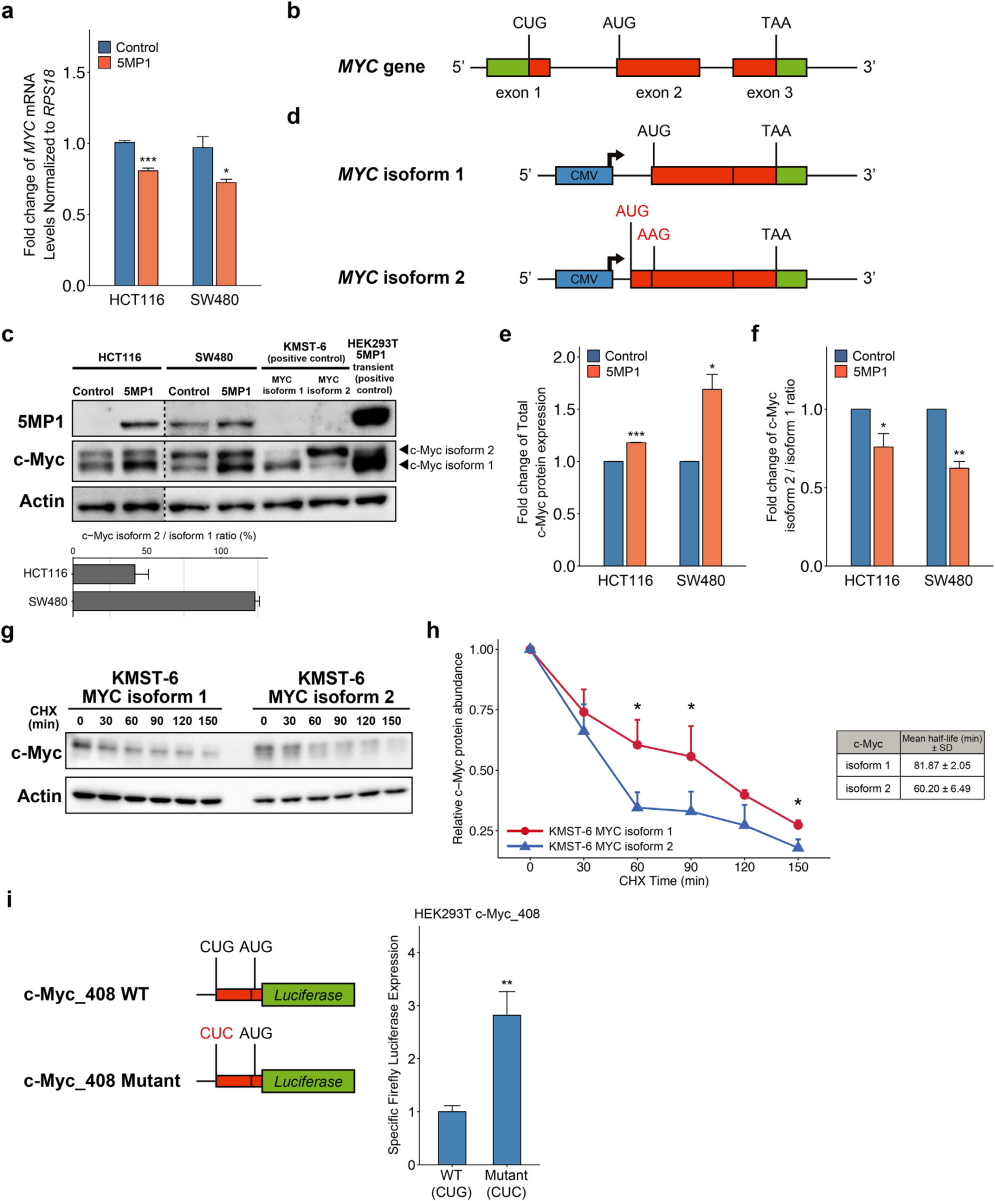
5MP1 accumulates stable AUG-initiated c-Myc protein. (a) Fold change in *MYC* mRNA expression levels measured by qRT-PCR in control cells and 5MP1-overexpressed cells, normalized to *RPS18* mRNA expression. (b) Map of the *MYC* gene. Exons 1–3 are represented by boxes, with the coding region filled with orange. Translational start codons and stop codons are indicated by black bars. (c) Western blot of 5MP1 and c-Myc in control and 5MP1-overexpressed cells. KMST-6 and HEK293T were used as positive controls for c-Myc and 5MP1, respectively. c-Myc isoforms 1 and 2 are indicated by arrowheads. Actin was used as the loading control (upper). Bar plots represent c-Myc isoform 2 to isoform 1 protein ratios in HCT116 control cells and SW480 control cells (lower). (d) Map of AUG-initiated *MYC* isoform (isoform 1) and CUG-initiated *MYC* isoform (isoform 2) lentiviral vectors. Blue boxes represent CMV promotor sequence. Mutated translational start codons are represented by red letters. (e) Fold change in c-Myc protein levels normalized to actin in control cells and 5MP1-overexpressed cells. (f) Fold change in c-Myc isoform 2 to isoform 1 protein ratios normalized to actin in control cells and 5MP1-overexpressed cells. (g) Cycloheximide (CHX) chase assay of c-Myc isoforms 1 and 2 in KMST-6 cells. KMST-6 cells stably expressing c-Myc isoform 1 or isoform 2 were treated with 50 μM CHX for the indicated times. The c-Myc protein levels were analyzed by Western blot with actin as a loading control. (h) Relative c-Myc levels normalized to actin remaining after CHX treatment were quantified and graphed as the percent (left). Summary of half-life for each c-Myc isoform (right). (i) Firefly luciferase expression levels of HEK293T cells transfected with WT and mutant c-Myc_408 Firefly luciferase plasmids and control Renilla luciferase plasmid. Firefly/Renilla ratio of mutant c-Myc_408 was compared to the value with WT c-Myc_408. *n* = 4 from two independent experiments. Data represent the mean ± SD of three independent experiments. n.s., not significant; (*) p < 0.05; (**) p < 0.01; (***) p < 0.001. (For interpretation of the references to colour in this figure legend, the reader is referred to the web version of this article.)

We next addressed the mechanism by which 5MP1 induces c-Myc protein expression. qRT-PCR showed that *MYC* mRNA expression levels are even significantly down-regulated in 5MP1-transfected CRC cells (Fig. 4a), suggesting that 5MP1 regulates translation, but not transcription, of *MYC* mRNA. c-Myc has two different isoforms, the shorter AUG-initiated isoform (isoform 1) and longer CUG-initiated isoform (isoform 2), which are translated from the AUG codon in exon2 and the CUG codon in exon1, respectively (Fig. 4b) [[62], [63], [64]]. Importantly, we recently showed that 5MP1 inhibits translation from non-AUG codons and thereby decreases the proportion of the CUG vs AUG-initiated c-Myc isoforms [31]. In agreement with this finding, our immunoblot analysis indicates a higher expression of the larger, presumably CUG-initiated c-Myc isoform (isoform 2) relative to the regular, smaller isoform (isoform 1) in HCT116 and SW480 with lower 5MP1 expression (Fig. 4c, mean ± SD; 41.9 ± 9.1 and 123.0 ± 3.1% isoform2/isoform1 ratio, respectively; *n* = 3). To identify the two isoforms, we used lysates prepared from stable KMST-6 fibroblast cell lines each expressing c-Myc isoform 1 or 2 (Fig. 4d) as positive controls (To achieve equal expression of isoform 1, we altered the CUG start codon of isoform 2 to AUG and its first AUG codon to AAG). Furthermore, stable 5MP1 transfection not only significantly decreased the CUG/AUG translation ratio in the CRC cells, but also increased the total c-Myc protein abundance (Fig. 4e and f). These results strongly suggest that 5MP1 promotes c-Myc expression by enhancing AUG-initiated translation of *MYC* transcript.

We noted that the CUG/AUG initiation ratios of 41.9–123.0% observed in *5MP1*-limited CRC are much higher than the ratio of ~5% previously observed with a dual luciferase assay in HEK293T [31]. However, HEK293T is replete with 5MP1/5MP2 (K.A., personal observation). Moreover, as shown in Fig. S5, two independent ribosome profiling studies of lactimidomycin (LTM)-treated HCT116 displayed even higher ribosome occupancy at the c-Myc CUG codon than at its AUG codon [65,66] (Fig. S5a and b). Furthermore, the ribosome occupancy at the AUG codon is increased by the knock-down of eIF1 with a role in non-AUG translation repression similar to 5MP1 [65] (Fig. S5c). These findings are consistent with the high CUG/AUG translation ratio observed in the *5MP1*-limited CRC cell lines.

To examine the mechanism of the c-Myc protein accumulation, especially its isoform 1, we examined two models; 1) Isoform 1 is more stable than isoform 2. 2) Upstream CUG initiation inhibits downstream AUG initiation. To address the first model, we conducted the cycloheximide chase assay to elucidate the differences in stability between isoforms 1 and 2 using the KMST-6 cells expressing these isoforms. Strikingly, the half-life of isoform 1 is significantly longer than that of isoform 2 (Fig. 4g and h). To examine the second model, we used a firefly luciferase reporter plasmid carrying the 5’ UTR of c-Myc, in which the luciferase reading frame was fused to c-Myc AUG start codon (cMyc_408, WT) [31]. The alteration of c-Myc CUG start codon to a CUC codon increased the specific firefly expression by ~3-fold (Fig. 4i), indicating a strong block of downstream AUG initiation by the CUG codon. This last result suggests that 5MP1 increases the proportion of isoform 1, not only by inhibiting isoform 2 initiation at CUG but also by enhancing isoform 1 initiation through alleviating the block by the former. We suggest that the two mechanisms are combined to ensure enhanced protein expression of c-Myc isoform 1. The extra N-terminal peptide added by CUG initiation decreases the stability of the c-Myc isoform 2 either directly or indirectly by affecting the co-translational folding of the body of c-Myc protein. In addition to enhanced AUG initiation due to repressed CUG initiation, the isoform 1 abundance may increase through decrease in the abundance of isoform 2, which may interact with isoform 1 and thereby decrease its stability, prior to their binding to the heterodimer partner, Max [67]. Differentiating these possibilities is an important subject of further studies.

**Fig. 5:**
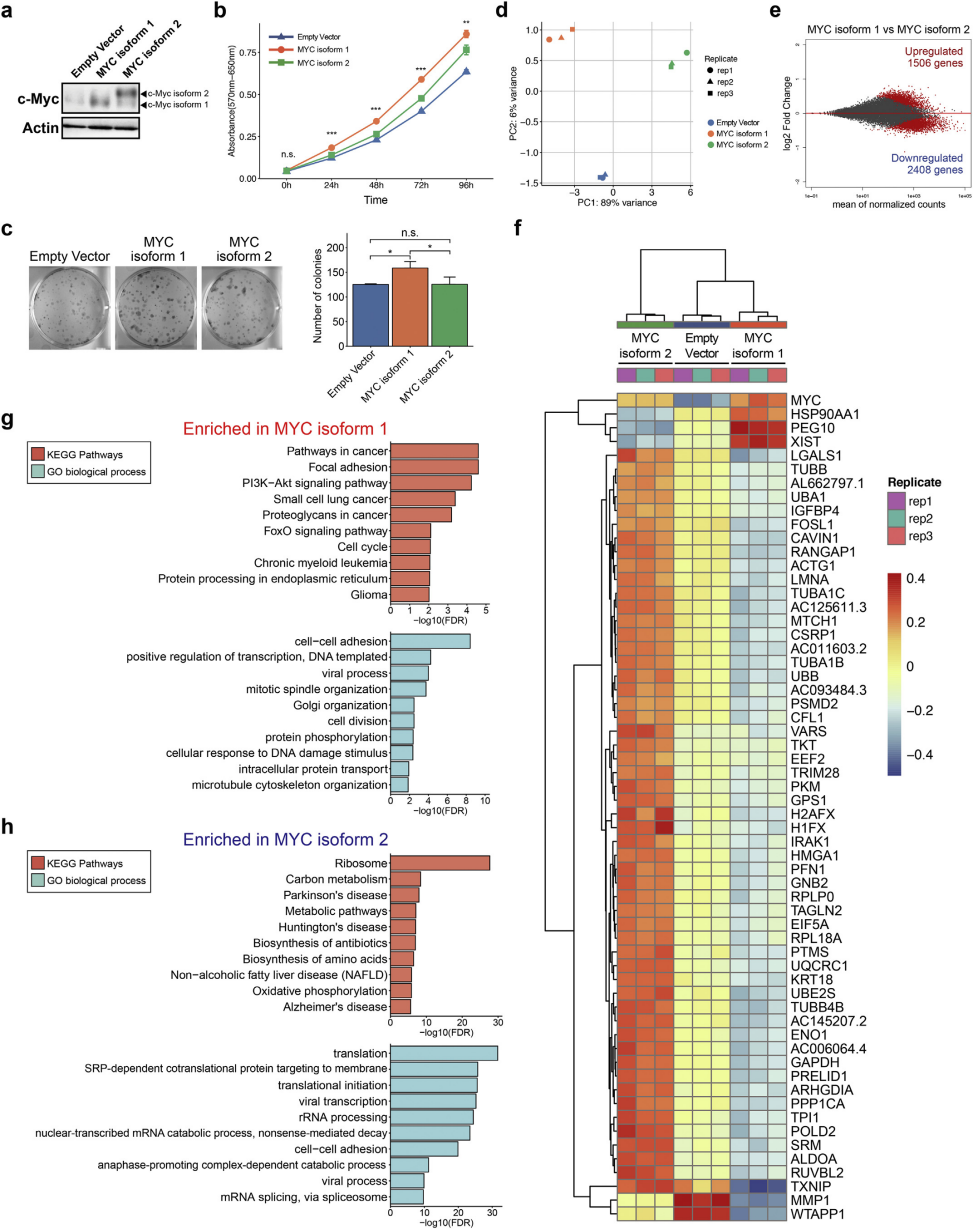
The differential effect of AUG- and CUG-initiated c-Myc isoforms (isoforms 1 and 2) against phenotypes and transcriptome. (a) Western blot of c-Myc in the indicated KMST-6 stable cell lines. Actin was used as a loading control. c-Myc isoforms 1 and 2 are indicated by arrowheads. (b) Proliferation curve of the indicated KMST-6 stable cell lines measured by MTT assays. (c) Representative images (left) and quantification of the colonies (right) of the indicated KMST-6 stable cell lines at day 14. (d) Principal component analysis of the indicated KMST-6 stable cell lines. Genes with zero counts across all samples were excluded from the analysis. (e) MA-plot of differentially expressed genes (DEGs) between the indicated KMST-6 stable cell lines. DEGs are represented as red dots. The cutoff of DEGs was determined as FDR < 0.01 (likelihood ratio test). The numbers of significantly up- or downregulated genes are shown. (f) Hierarchical clustering of the top 60 DEGs (FDR < 0.01, likelihood ratio test) among the indicated KMST-6 stable cell lines. Regularized log2 expression values are row-mean subtracted. (g,h) Gene Ontology (GO) analysis of significantly upregulated genes in c-Myc isoform 1 (g) and isoform 2 (h). Top 10 significantly enriched KEGG pathways and GO biological process are shown (FDR < 0.05). Data represent the mean ± SD of three independent experiments. n.s., not significant; (*) p < 0.05; (**) p < 0.01; (***) p < 0.001. (For interpretation of the references to colour in this figure legend, the reader is referred to the web version of this article.)

**Fig. 6:**
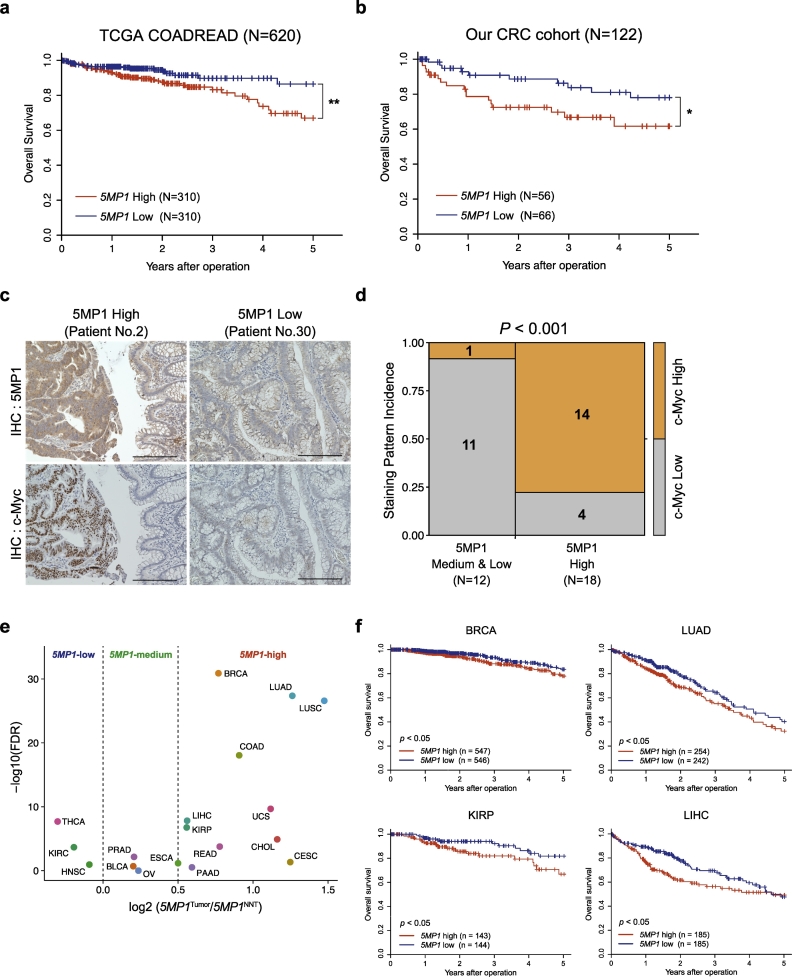
Clinical relevance of 5MP1 expression in CRC patients and pan-cancer analysis of 5MP1. (a,b) Kaplan-Meier curves for overall survival of CRC patients in TCGA COADREAD dataset (*N* = 620) (a) and our cohort (*N* = 122) (b) classified according to the *5MP1* mRNA expression levels in tumor tissues. *P*-values were calculated using the log-rank test. (*) p < 0.05; (**) *p* < 0.01. (c) Representative images of immunohistochemical staining for 5MP1 and c-Myc in CRC tissues; 5MP1 High (left) and 5MP1 Low (right). Scale bars, 200 μm. (d) Mosaic plot summarizing 5MP1 and c-Myc expression in CRC tissues for the indicated number of CRC patients (*N* = 30). The p-value for the association between the parameters was calculated via Fisher's exact test. (e) Pan-cancer analysis of *5MP1* expression in the TCGA dataset. The log2-fold change in *5MP1* mRNA expression in tumor tissues compared to that in NNT and the -log10 (FDR) in each tumor types are shown. Dashed lines show the cut-off lines of *5MP1*-low, *5MP1*-medium and *5MP1*-high tumor types. (f) Kaplan-Meier overall survival curves showing that *5MP1* is positively correlated with poor prognosis in the indicated TCGA datasets. P-values were calculated using the log-rank test. BLCA, bladder urothelial carcinoma; BRCA, breast invasive carcinoma; CESC, cervical and endocervical cancers; CHOL, cholangiocarcinoma; COAD, colon adenocarcinoma; ESCA, esophageal carcinoma; HNSC, head and neck squamous cell carcinoma; KIRC, kidney renal clear cell carcinoma; KIRP, kidney renal papillary cell carcinoma; LIHC, liver hepatocellular carcinoma; LUAD, lung adenocarcinoma; LUSC, lung squamous cell carcinoma; OV, ovarian serous cystadenocarcinoma; PAAD, pancreatic adenocarcinoma; PRAD, prostate adenocarcinoma; READ, rectal adenocarcinoma; THCA, thyroid carcinoma; UCS, uterine carcinosarcoma.

**Table 1:** *5MP1* mRNA expression and clinicopathological factors in CRC patients.

Factors	5MP1 High (N = 56)	5MP1 Low (N = 66)	P-Value
Age			0.438
<65	19	18	
≧65	37	48	
Gender			0.581
Male	36	39	
Female	20	27	
Histology			0.752
Well or moderately differentiated	52	60	
Poorly differentiated	4	6	
Depth of invasion			0.141
M, SM, MP	10	20	
SS, SE, SI	46	46	
Lymph node metastasis			0.856
Absent	29	36	
Present	27	30	
Lymphatic invasion			1.000
Absent	32	37	
Present	24	29	
Venous invasion			0.034⁎
Absent	32	50	
Present	24	16	
Distant metastasis			0.018⁎
Absent	51	66	
Present	5	0	
UICC TNM stage			1.000
I/II	29	35	
III/IV	27	31	

M, mucosa; SM, submucosa; MP, muscular propria; SS, subserosa; SE, serosal invasion; SI, invasion to adjacent organs; UICC TNM stage, Union for International Cancer Control tumor-node-metastasis stage.

⁎ Statistically significant.

### 3.6 The distinct effects of AUG- and CUG-initiated c-Myc isoforms (isoform 1 and 2) on proliferation and the transcriptome

To explore the functional differences between c-Myc isoforms 1 and 2, we examined the proliferation of the KMST-6 cells expressing these isoforms in vitro (Fig. 5a). This cell line does not express c-Myc, and therefore offers a clean system to evaluate the biological effect of the expressed isoforms [68]. Notably, we found that isoform 1 significantly and more strongly promoted proliferation and colony formation than did isoform 2 and the control (Fig. 5b and c). Thus, the isoform 1 induced by 5MP1 is a more oncogenic, proliferative form. These results motivated us to investigate the differences in transcriptional targets between the two c-Myc isoforms via RNA-seq. Remarkably, the principal component analysis (PCA) and hierarchical clustering showed that the transcriptional landscape of isoform 1 is different from that of isoform 2, suggesting different transcriptional targets for these two isoforms (Fig. 5d and S6a). This analysis revealed 3914 differentially expressed genes (DEGs) between isoform 1, isoform 2, and the control. We found 1506 genes that were significantly upregulated and 2408 genes that were significantly downregulated in cells expressing isoform 1 compared to those expressing isoform 2 (Benjamini-Hochberg adjusted *p*-value <0.01, likelihood ratio test) (Fig. 5e and S6b). Interestingly, several c-Myc downstream target genes such as PEG10 and TXNIP [69,70] were conversely regulated by these two isoforms (Fig. 5f and S6c). To determine the pathways that were enriched differentially between these two isoforms, we performed GO analysis of DEGs. Remarkably, several oncogenic pathways and cell-cycle-related pathways were significantly enriched in cells expressing isoform 1, whereas pathways involved in ribosome biogenesis and metabolic pathways such as carbon metabolism and amino-acid biogenesis were enriched in cells expressing isoform 2 (Fig. 5g and h). These results indicate that isoform 1, not isoform 2, is a proliferative isoform at the phenotypic levels and that these two isoforms have different transcriptional targets. In summary, c-Myc isoform 1 (AUG-initiated) may facilitate tumor growth not only by its stability but also by its distinct transcriptional targets.

### 3.7 High 5MP1 expression levels predict poor prognosis in patients with CRC

To elucidate the clinical significance of *5MP1* expression in patients with CRC, we performed a survival analysis in two independent cohorts, the TCGA COADREAD dataset (*N* = 620) and CRC patient dataset from our hospital and affiliated facilities (*N* = 122). Importantly, the high-*5MP1*-expression group exhibited significantly poorer overall survival (OS) than the low-expression group in both TCGA and our dataset (log-rank test, *p* = 0.008 and *p* = 0.04, respectively) (Fig. 6a and b). In the clinicopathological analysis in our dataset, high *5MP1* expression was positively correlated with venous invasion and distant metastasis (Table 1). These data imply that the high expression of *5MP1* clinically contributes to the malignant phenotype of CRC. Interestingly, mRNA expression levels of eIF5-mimic protein 2 (*5MP2*), also known as basic leucine zipper and W2 domains 1 (*BZW1*) and a paralog of *5MP1* [23], had no significant effect on the survival of patients with CRC (Fig. S7a). Notably, the high-*eIF5*-expression group exhibited a better prognosis than the low-expression group (Fig. S7b). Furthermore, the *eIF5* DNA copy is significantly more often deleted, and eIF5 expression is significantly lower in CRC tissues than in NNT, regardless of primary sites or tumor grades (Fig. S7c and d). Consistent with these findings, immunohistochemical staining and LC/MS dataset of TCGA revealed the low expression levels of eIF5 in CRC tissues (Fig. S7e and f). These data suggest that the translational reprogramming caused by 5MP1 has a crucial impact on the prognosis of patients with CRC, in contrast to that by 5MP2 or eIF5. Furthermore, we explored the correlation between 5MP1 and c-Myc protein expression in CRC tissues. Immunohistochemical staining of tumor lesions showed that 5MP1 and c-Myc protein expression is positively correlated (Fisher's exact test, *P* < 0.001) (Fig. 6c and d). These clinical data strongly support our experimental findings that 5MP1 up-regulates c-Myc expression and promotes its oncogenic function. Additionally, the overexpression of *5MP1* is widely observed, and the high expression group showed poor prognosis in various types of cancer (Fig. 6e and f), suggesting that 5MP1 may have oncogenic functions in various cancer, as well as in CRC.

## 4 Discussion

In this study, we discovered that 5MP1 is a novel oncogene in CRC and that its gain-of-function via amplification of its DNA copy number contributes to the tumor growth of CRC and poor prognosis in CRC patients. Furthermore, our studies showed that 5MP1 reprograms the translation initiation of c-Myc oncogene and increases the AUG-initiated c-Myc isoform, which is more oncogenic than the CUG-initiated counterpart. To the best of our knowledge, this is the first study which demonstrates that the reprogramming of translation initiation affects not only the malignant phenotypes of CRC but also the clinical outcome of CRC patients.

Previous studies reported that non-AUG translation alters protein functions in cancer. For example, the tumor suppressor protein WT1 changes its role to an oncogenic protein, when its translation is initiated from an upstream CUG start codon [71]. In contrast, the tumor suppressor PTEN becomes more tumor-suppressive when it is translated from an upstream alternative CUG start codon, generating the secretory variant PTEN-Long. Since PTEN-Long antagonizes PI3K signaling and induces tumor cell death as an exogenous agent, recombinant PTEN-Long protein is expected to have therapeutic uses [72,73]. However, none of these studies identified the regulatory factor responsible for non-AUG translation of these target genes. In this regard, Sendoel et al. identified non-canonical translation initiation factor eIF2A that promotes translation from a specific codon, CUG, as driver of tumor initiation in the SOX2-induced skin squamous cell carcinoma tumorigenesis mice model [8]. However, the relationship between eIF2A-mediated non-AUG translation and functional changes of the oncogenes, as well as its clinical impact, remain to be elucidated. Moreover, eIF2A is not prognostic for CRC, breast cancer or lung cancer in TCGA and CRC in our dataset (K.S. and K.A., unpublished data). These apparent discrepancies might be explained by the contextual importance of other types of non-AUG codons. For example, GUG initiates translation of eIF4G2/NAT1 that plays an important role in stem cell differentiation [74]. 5MP1 can repress *NAT1* translation [31]. With its ability to regulate translation from all types of non-AUG codons, 5MP1 is therefore considered to be an excellent therapeutic target of CRC.

5MP increases the accuracy of translation initiation by competing with eIF5. Accordingly, the balance between the expression of 5MP and eIF5 determines the non-AUG initiation rate [23,31]. siRNA-induced knockdown of eIF5 and eIF1 attenuated the viability of HCT116, in agreement with their essential role in translation initiation [52]. Ribosome profiling of HCT116 derivatives knocked down for eIF1 demonstrated an increase in non-AUG translation genome-wide in CRC [65]. The present study paves the way towards understanding how 5MP reprograms genome-wide translation in CRC through regulating non-AUG translation, using c-Myc mRNA as a model case. Interestingly, we previously noted that high expression of *5MP1*, but not that of *5MP2*, correlates with poorer prognosis of breast and lung cancer patients, while in contrast, high expression of *eIF5* correlates with their better prognosis [31]. Moreover, eIF5 on chromosome 14q is frequently deleted [20], while 5MP1 on chromosome 7p is amplified in CRC, as described above. These observations strongly suggest that the balance between 5MP1 and eIF5 expression is disrupted in disfavor of non-AUG translation in CRC.

Here, we identified c-Myc as the target of translation reprogramming by 5MP1. We observed that 5MP1 regulates the translation initiation of c-Myc and better induces the AUG-initiated c-Myc isoform, which promotes cell proliferation, compared to the CUG-initiated isoform. In agreement with this finding, the functional differences between the two isoforms of c-Myc had been previously discussed. The CUG-initiated c-Myc isoform is suggested to have additional DNA-binding capabilities and its overexpression, but not that of the AUG-initiated isoform, inhibited cell growth [64]. Inactivation of the CUG-initiated isoform is observed in lymphoma cells, suggesting that the AUG-initiated isoform could have an advantage in tumor growth [63]. c-Myc is associated with many cellular processes such as stemness, proliferation, and metabolism [67,75], and is one of the most common and frequently activated oncogenic transcription factors in various types of cancer [54]. Thus, the regulation of c-Myc by 5MP1-induced translational reprograming is likely to have a crucial impact on tumor progression. In addition to c-Myc, we previously reported that 5MP1 induces the translation of the oncogenic transcription factor ATF4 through modulating the frequency of ribosome re-initiation [25,48]. Our ribosome profiling studies also suggested that there would be various other targets involved in oncogenic transcription, whose translation is controlled by 5MP1 through non-AUG translation [31]. Further study is warranted to identify and characterize 5MP1 downstream target genes and to elucidate their biological and clinical significance in cancer.

Tumor heterogeneity is a major cause of the therapeutic difficulty of cancer due to the presence of multiple subclones boosting adaptation to pharmacologic interventions [76]. It is noteworthy that the amplification of 5MP1 is widely observed not only in tumors but also in adenoma and is ubiquitously observed in multiple regions of CRC, as we have reported previously [17,18]. This observation along with its apparent purifying selection during CRC development suggests that the overexpression and resulting gain-of-function of 5MP1 is a driver event spatiotemporally shared in the cancer evolution of CRC. Therefore, 5MP1 deserves much better attention as a potential therapeutic target in CRC to overcome the therapeutic resistance conferred by tumor heterogeneity.

The following are the supplementary data related to this article.

Fig. S1 5MP1 on chromosome 7p is amplified in CRC. Related to Figure 1. (a) Arm level copy number alterations in CRC tissues from the TCGA COADREAD dataset. The copy number of each chromosome arm in Non-neoplastic tissues of colorectal mucosa (NNT, left) and CRC tissues (right) are shown. (b) Schematic diagram of the strategy for detection of 5MP1. (c) *5MP1* copy number in CRC tissues and NNT in TCGA dataset. P represents *p*-values from the two-sided Mann-Whitney *U* test. (d) Violin plots of *5MP1* mRNA expression levels in CRC tissues and NNT in the TCGA COADREAD dataset. *P*-values were calculated by pairwise comparisons using the Mann-Whitney U test with Bonferroni posttest. LG, Low Grade; HG, High Grade; n.s., not significant. (e) GSEA with a significant enrichment score showing a c-Myc target geneset and cell-cycle-related genesets that are positively correlated with *5MP1* expression in the TCGA dataset. NES, normalized enrichment score. Fig. S1

Fig. S2 Distributions of dN/dS ratios in TCGA CRC dataset. Related to Figure1. A histogram of dN/dS ratios per gene for missense mutations estimated by dNdScv in TCGA CRC dataset (*N* = 561). *5MP1* and known driver genes in CRC are indicated. Fig. S2

Fig. S3 Expression levels of *5MP1* and eIF5 and the effect of 5MP1 overexpression in CRC cell lines. Related to Figure 2. (a) Relative *5MP1* mRNA expression levels measured by qRT-PCR in CRC cel lines, normalized to *GAPDH* mRNA expression. Data represent the mean ± SD of three independent experiments. (b) Proliferation curves of 5MP1-overexpressed HCT116 cells and control cells measured by MTT assays. (***) *p* < 0.001; Dunnet-test was used to adjust for multiple comparisons. Data represent mean ± SEM (*n* = 9 for each group). (c) Representative images of oncospheres derived from 5MP1-overexpressed HCT116 cells and control cells. (d) Western blot of eIF5 in control cells and 5MP1-overexpressed cells. Actin was used as the loading control (e) Western blot of eIF5 in control cells and siRNA-mediated 5MP1-knockdown cells. Actin was used as the loading control. Fig. S3

Fig. S4 5MP1 induces c-Myc-mediated cell cycle progression. Related to Figure. (a) Cell cycle assay of siRNA-mediated 5MP1-knockdown cells. Bar graphs represent the cell distribution in each cell cycle phase. (b) GSEA with a significant enrichment score showing a cell-cycle-related geneset that are positively correlated with *5MP1* expression in 5MP1-overexpressed cells. NES, normalized enrichment score. (c) Schematic of SCADS inhibitor screening analysis. (d) Representative data showing cell growth of control cells and 5MP1-overexpressed cells following treatment with each listed compound targeting the molecules displayed. (e) Apoptosis assay of 5MP1-overexpressed HCT116 cells. Bar graphs represent the cell distribution in late apoptosis (Annexin V-FITC+/propidium iodide [PI]+). Data represent the mean ± SD. (*) *p* < 0.05; (**) *p* < 0.01; (***) *p* < 0.001. Fig. S4

Fig. S5 Ribosome-protected profiles of c-Myc start codons in lactimidomycin (LTM)-treated HCT116 cells. Related to Figure 4. (a-c) The ribosome occupancy at CUG and AUG start codons of the *MYC* mRNA derived from LTM-treated HCT116 cells is shown as green peaks. All data of the indicated studies was obtained from the Trips-viz website (https://trips.ucc.ie/). Fig. S5

Fig. S6 Transcriptional profiling of KMST-6 cells expressing AUG- and CUG-initiated c-Myc isoforms (isoform 1 and 2). Related to Figure5. (a) Transcriptomic distances of the KMST-6 stable cell lines expressing the indicated lentiviral vectors. (b) MA-plot of differentially expressed genes (DEGs) between the indicated KMST-6 stable cell lines. DEGs are represented as red dots. The cutoff of DEGs was determined as FDR < 0.01 (Likelihood ratio test). The numbers of significantly up- or downregulated genes are shown. (c) Hierarchical clustering of the top 50 upregulated genes (FDR < 0.01, likelihood ratio test) in isoform 1 (left) isoform 2 (right) compared to isoform 2 and isoform 1, respectively. Regularized log2 expression values are row-mean subtracted. (For interpretation of the references to color in this figure legend, the reader is referred to the web version of this article.) Fig. S6

Fig. S7 Clinical relevance of 5MP2 and eIF5 expression levels in CRC. Related to Figure6. (a-b) Kaplan-Meier curves for the overall survival of CRC patients in the TCGA COADREAD dataset (*N* = 620), classified according to the *5MP2* mRNA expression levels (a) and *eIF5* mRNA expression levels (b) in tumor tissues. *P*-values were calculated using the log-rank test. (c) *eIF5* copy number in CRC tissues and non-neoplastic tissues of colorectal mucosa (NNT) in TCGA dataset. P represents *p*-values from the two-sided Mann-Whitney *U* test. (d) Violin plots of *eIF5* mRNA expression levels in CRC tissues and NNT in the TCGA COADREAD dataset. P-values were calculated by pairwise comparisons using the Mann-Whitney U test with Bonferroni posttest. LG, Low Grade; HG, High Grade; n.s., not significant. (e) Representative images of immunohistochemical staining for eIF5 in CRC tissues (upper). Proportions of eIF5 levels in tumor tissues and NNT are shown using three-stage staining score (lower). T, Tumor; N, NNT; scale bars, 200 μm. (f) Violin plots of eIF5 protein expression levels in CRC tissues and NNT in the TCGA COADREAD dataset. *P* represents p-values from the two-sided Mann-Whitney U test. Fig. S7

Table S1 Resources used in this study. Table S1

## Funding sources

This work was supported in part by the following grants and foundations: Japan Society for the Promotion of Science [15H0912, 15H05707, 16K07177, 16K10543, 16K19197, 17K16454, 17K16521, 17K10593 and 17K19608]; OITA Cancer Research Foundation; Priority Issue on Post-K computer [hp170227, hp160219]; Eli Lilly Japan K.K. Grant; Grants from National Institutes of Health [R15 GM124671 to K.A.]; Research grant from National Science Foundation [MCB 1412550 to K.A.]; An Innovative Award from KSU Terry Johnson Cancer Center [to K.A.]; Faculty Development Awards from KSU [to K.A.]. S. G. and M. T. received K-INBRE scholarship [National Institutes of Health, P20GM103418].

## Declaration of interests

The authors declare no competing interests.

## Author contributions

K.S., T.M. and K.A. designed the study. K.S., T.M., S.G. and M.T. performed the experiments. K.S., Q.H. and A.N. performed bioinformatics analyses. T.M., Q.H., Y.K., H.E. and K.M. collected surgically resected human samples. T.T. performed histological analysis. K.S. wrote the original draft of the manuscript. K.S., T.M., K.A. and K.M. reviewed and edited the manuscript. T.N., K.A., and K.M. supervised the research.
