# Supplementary material for: Novel oncogene 5MP1 reprograms c-Myc translation initiation to drive malignant phenotypes in colorectal cancer
Source: eBioMedicine. 2019 Jun 4;44:387–402. doi: 10.1016/j.ebiom.2019.05.058 (PMC6606960; doi:10.1016/j.ebiom.2019.05.058)
Supplement: Fig. S1 — 5MP1 on chromosome 7p is amplified in CRC. Related to Figure 1. (a) Arm level copy number alterations in CRC tissues from the TCGA COADREAD dataset. The copy number of each chromosome arm in Non-neoplastic tissues of colorectal mucosa (NNT, left) and CRC tissues (right) are shown. (b) Schematic diagram of the strategy for detection of 5MP1. (c) 5MP1 copy number in CRC tissues and NNT in TCGA dataset. P represents p-values from the two-sided Mann-Whitney U test. (d) Violin plots of 5MP1 mRNA expression levels in CRC tissues and NNT in the TCGA COADREAD dataset. P-values were calculated by pairwise comparisons using the Mann-Whitney U test with Bonferroni posttest. LG, Low Grade; HG, High Grade; n.s., not significant. (e) GSEA with a significant enrichment score showing a c-Myc target geneset and cell-cycle-related genesets that are positively correlated with 5MP1 expression in the TCGA dataset. NES, normalized enrichment score. [file mmc1.pdf]

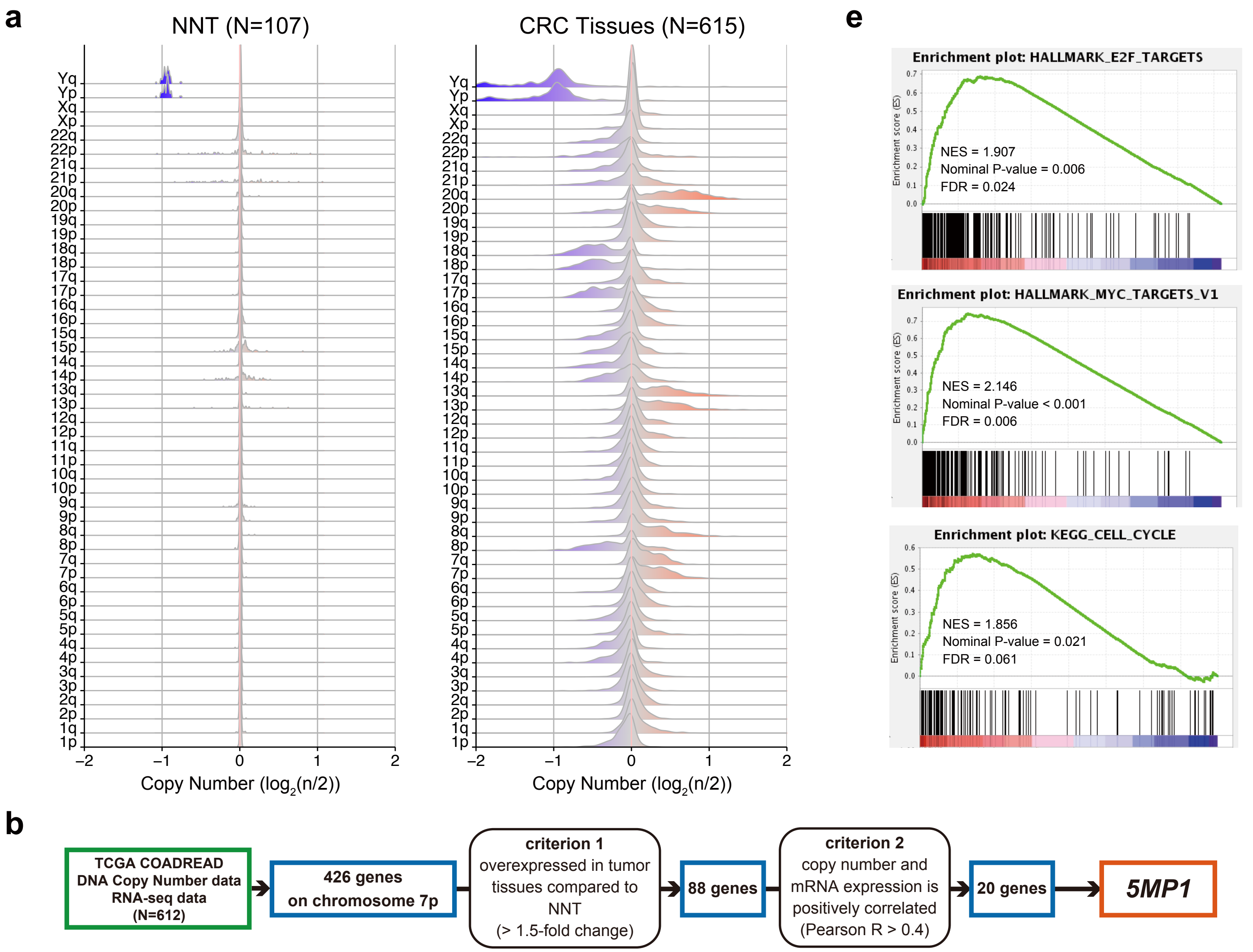

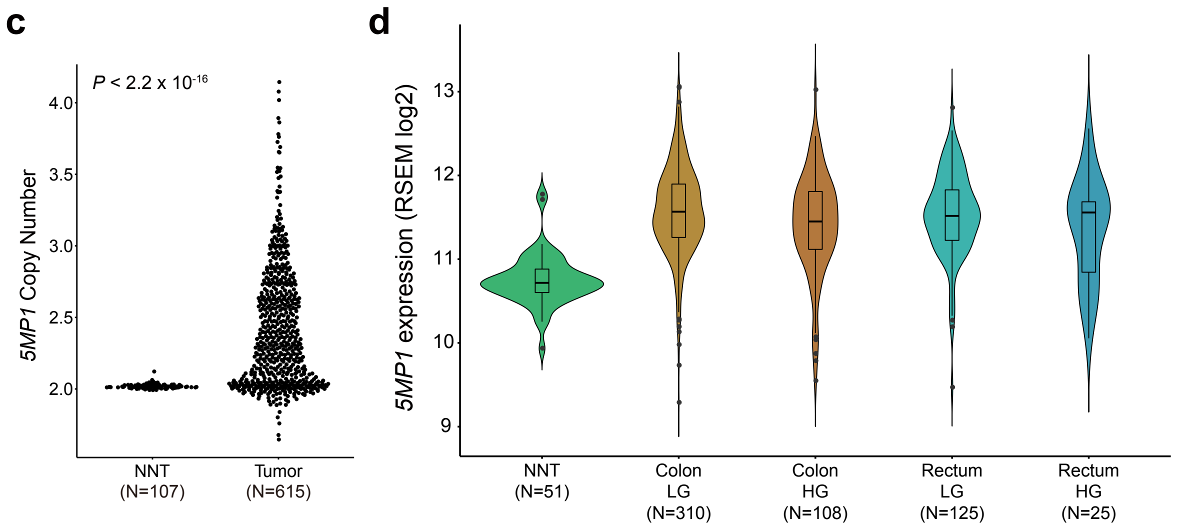

Comparison	P-Value
NNT vs Colon LG	< 2.2×10^{-16}
NNT vs Colon HG	2.2×10^{-12}
NNT vs Rectum LG	< 2.2×10^{-16}
NNT vs Rectum HG	7.6×10^{-4}
Colon LG vs Colon HG	n.s.
Colon LG vs Rectum LG	n.s.
Colon LG vs Rectum HG	n.s.
Colon HG vs Rectum LG	n.s.
Colon HG vs Rectum HG	n.s.
Rectum LG vs Rectum HG	n.s.

Figure S1. 5MP1 on chromosome 7p is amplified in CRC. Related to Figure 1. (a) Arm level copy number alterations in CRC tissues from the TCGA COADREAD dataset. The copy number of each chromosome arm in Non-neoplastic tissues of colorectal mucosa (NNT, left) and CRC tissues (right) are shown. (b) Schematic diagram of the strategy for detection of 5MP1. (c) *5MP1* copy number in CRC tissues and NNT in TCGA dataset. P represents p-values from the two-sided Mann-Whitney U test.(d) Violin plots of *5MP1* mRNA expression levels in CRC tissues and NNT in the TCGA COADREAD dataset. P-values were calculated by pairwise comparisons using the Mann-Whitney U test with Bonferroni posttest. LG, Low Grade; HG, High Grade; n.s., not significant. (e) GSEA with a significant enrichment score showing a c-Myc target geneset and cell-cycle-related genesets that are positively correlated with *5MP1* expression in the TCGA dataset. NES, normalized enrichment score.
