# Supplementary material for: Novel oncogene 5MP1 reprograms c-Myc translation initiation to drive malignant phenotypes in colorectal cancer
Source: eBioMedicine. 2019 Jun 4;44:387–402. doi: 10.1016/j.ebiom.2019.05.058 (PMC6606960; doi:10.1016/j.ebiom.2019.05.058)
Supplement: Fig. S2 — Distributions of dN/dS ratios in TCGA CRC dataset. Related to Figure1. A histogram of dN/dS ratios per gene for missense mutations estimated by dNdScv in TCGA CRC dataset (N = 561). 5MP1 and known driver genes in CRC are indicated. [file mmc2.pdf]

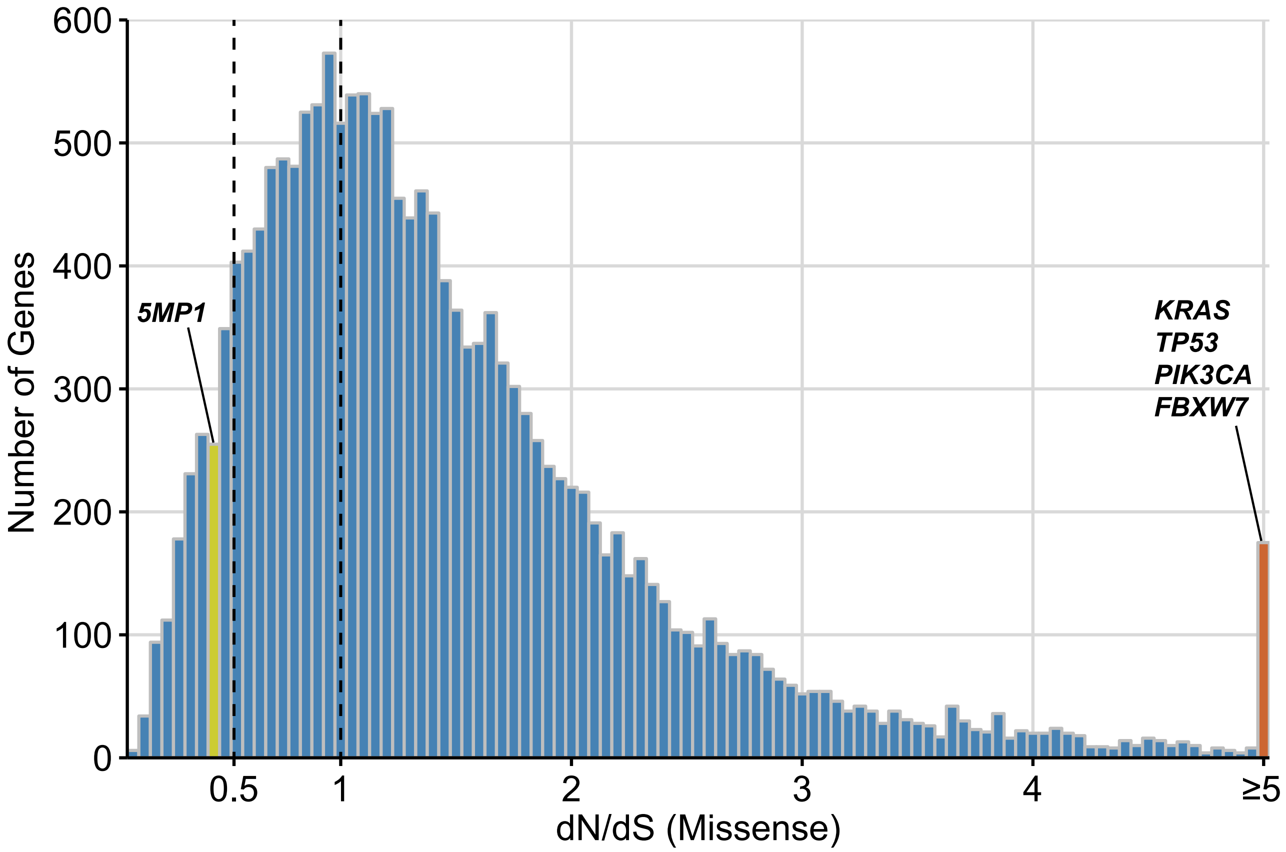

Figure S2. Distributions of dN/dS ratios in TCGA CRC dataset. Related to Figure1. A histogram of dN/dS ratios per gene for missense mutations estimated by dNdScv in TCGA CRC dataset (N=561). *5MP1* and known driver genes in CRC are indicated.
