# Supplementary material for: Novel oncogene 5MP1 reprograms c-Myc translation initiation to drive malignant phenotypes in colorectal cancer
Source: eBioMedicine. 2019 Jun 4;44:387–402. doi: 10.1016/j.ebiom.2019.05.058 (PMC6606960; doi:10.1016/j.ebiom.2019.05.058)
Supplement: Fig. S3 — Expression levels of 5MP1 and eIF5 and the effect of 5MP1 overexpression in CRC cell lines. Related to Figure 2. (a) Relative 5MP1 mRNA expression levels measured by qRT-PCR in CRC cel lines, normalized to GAPDH mRNA expression. Data represent the mean ± SD of three independent experiments. (b) Proliferation curves of 5MP1-overexpressed HCT116 cells and control cells measured by MTT assays. (***) p < 0.001; Dunnet-test was used to adjust for multiple comparisons. Data represent mean ± SEM (n = 9 for each group). (c) Representative images of oncospheres derived from 5MP1-overexpressed HCT116 cells and control cells. (d) Western blot of eIF5 in control cells and 5MP1-overexpressed cells. Actin was used as the loading control (e) Western blot of eIF5 in control cells and siRNA-mediated 5MP1-knockdown cells. Actin was used as the loading control. [file mmc3.pdf]

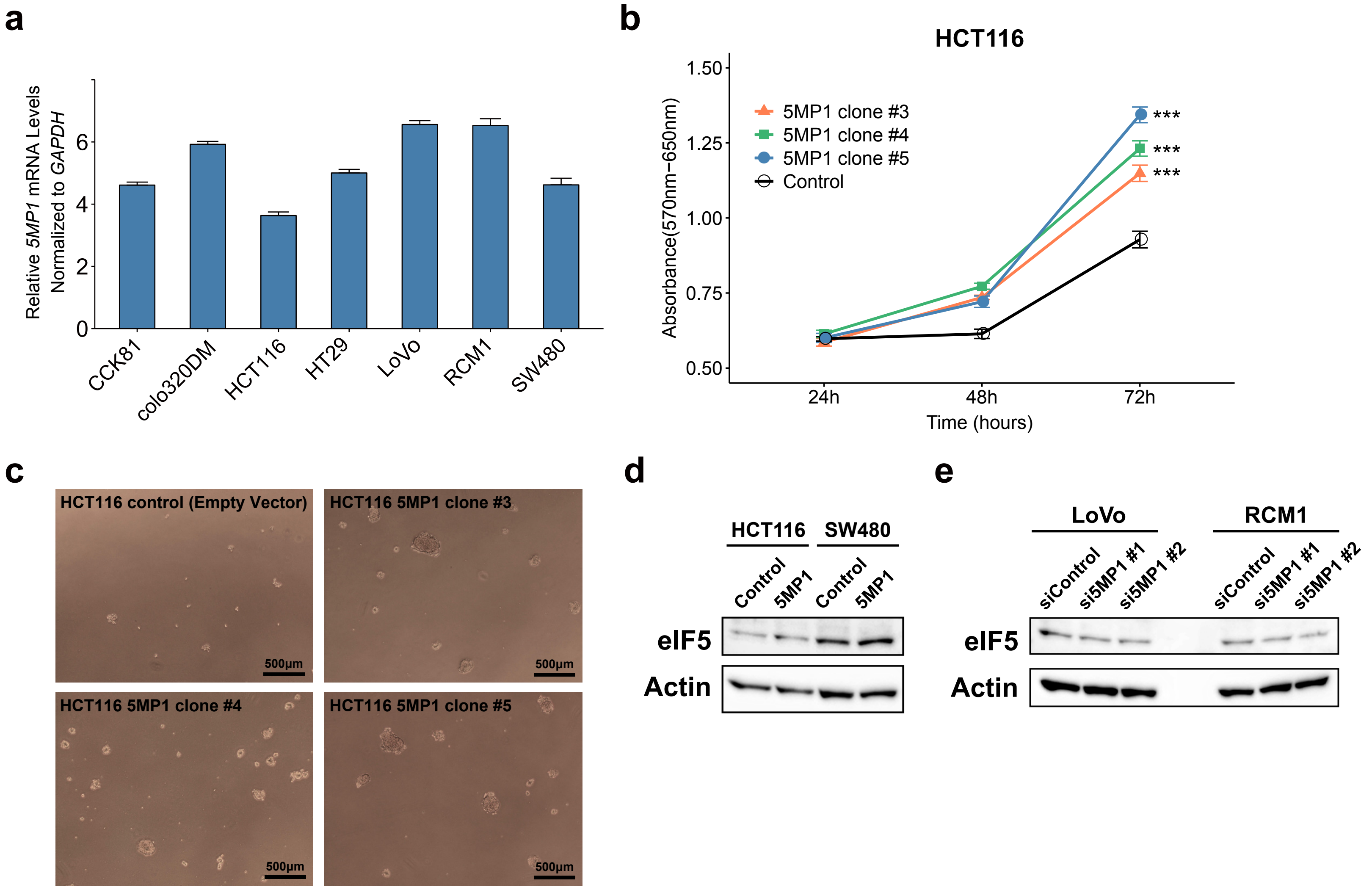


**Figure S3. Expression levels of *5MP1* and eIF5 and the effect of 5MP1 overexpression in CRC cell lines. Related to Figure2.** (a) Relative *5MP1* mRNA expression levels measured by qRT-PCR in CRC cel lines, normalized to *GAPDH* mRNA expression. Data represent the mean ± SD of three independent experiments. (b) Proliferation curves of 5MP1-overexpressed HCT116 cells and control cells measured by MTT assays. (***) $p<0.001$; Dunnet-test was used to adjust for multiple comparisons. Data represent mean ± SEM (n=9 for each group). (c) Representative images of oncospheres derived from 5MP1-overexpressed HCT116 cells and control cells. (d) Western blot of eIF5 in control cells and 5MP1-overexpressed cells. Actin was used as the loading control (e) Western blot of eIF5 in control cells and siRNA-mediated 5MP1-knockdown cells. Actin was used as the loading control.
