# Supplementary material for: Novel oncogene 5MP1 reprograms c-Myc translation initiation to drive malignant phenotypes in colorectal cancer
Source: eBioMedicine. 2019 Jun 4;44:387–402. doi: 10.1016/j.ebiom.2019.05.058 (PMC6606960; doi:10.1016/j.ebiom.2019.05.058)
Supplement: Fig. S4 — 5MP1 induces c-Myc-mediated cell cycle progression. Related to Figure. (a) Cell cycle assay of siRNA-mediated 5MP1-knockdown cells. Bar graphs represent the cell distribution in each cell cycle phase. (b) GSEA with a significant enrichment score showing a cell-cycle-related geneset that are positively correlated with 5MP1 expression in 5MP1-overexpressed cells. NES, normalized enrichment score. (c) Schematic of SCADS inhibitor screening analysis. (d) Representative data showing cell growth of control cells and 5MP1-overexpressed cells following treatment with each listed compound targeting the molecules displayed. (e) Apoptosis assay of 5MP1-overexpressed HCT116 cells. Bar graphs represent the cell distribution in late apoptosis (Annexin V-FITC+/propidium iodide [PI]+). Data represent the mean ± SD. (*) p < 0.05; (**) p < 0.01; (***) p < 0.001. [file mmc4.pdf]

a

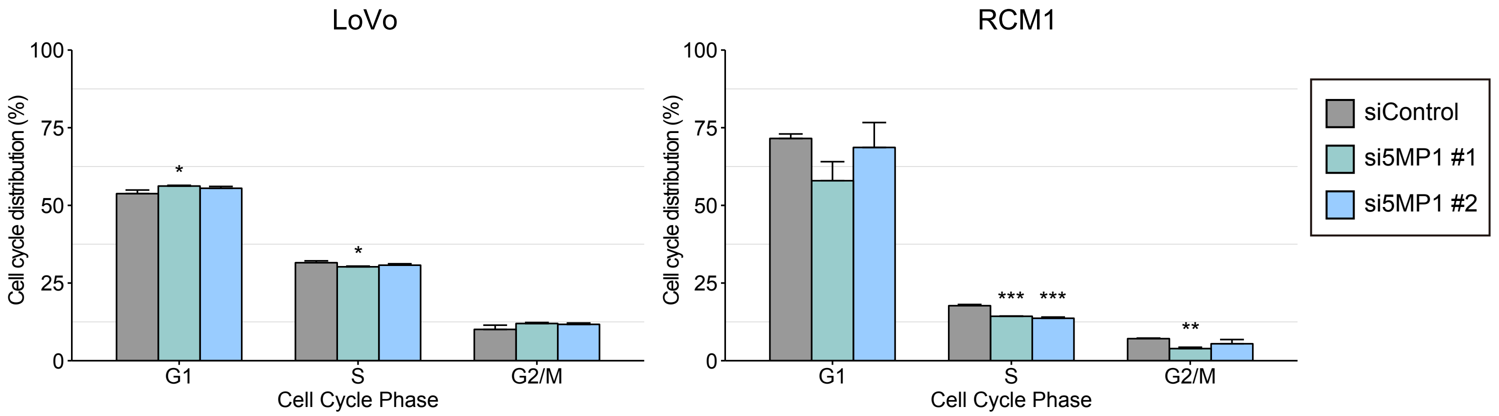

b

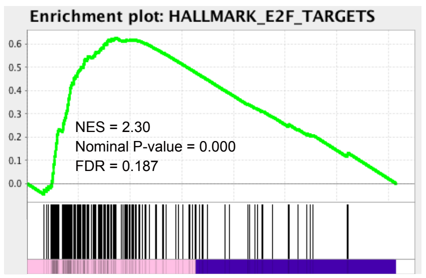

c

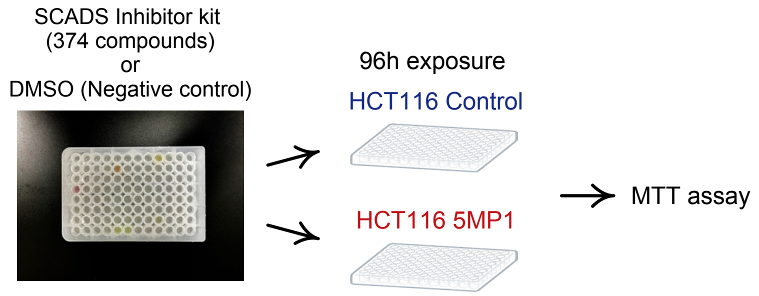

d

Targets		Compound	Cell Viability (Mean OD 570nm-650nm)	
			Control	5MP1
CDK	CDK2/CyclinA, CDK2/CyclinE, CDC2/CyclinB, CDK5/p35, MAPK	Olomoucine	1.234	0.949
	CDK2/CyclinA, CDK2/CyclinE, CDC2/CyclinB, CDK5/p35	Purvalanol A	1.334	0.985
	CDK1/CyclinB, CDK2/CyclinA, CDK2/CyclinE	Kenpaullone	1.414	1.040
	CDK1/CyclinB, CDK2/CyclinA, VEGF-R2, GSK3	CDK1/2 Inhibitor III	0.443	0.282
	CDK1/CyclinB, CDK5/p53, GSK3	Indirubin-3'-monoxime	1.285	1.096
	CDK1/CyclinB, CDK2/CyclinA, CDK5/p25, GSK3	GSK-3 inhibitor IX	1.418	0.961
CDK/Aurora	Aurora-A, Aurora-B, CDK1/CyclinB, CDK2/CyclinA, CDK2/CyclinE	JNJ-7706621	1.301	0.927
Aurora	Aurora-A	ENMD-2076	1.160	0.887
	Aurora-A	MLN8237	1.135	0.850
	Aurora-A	Aurora kinase inhibitor III	1.269	0.982

e

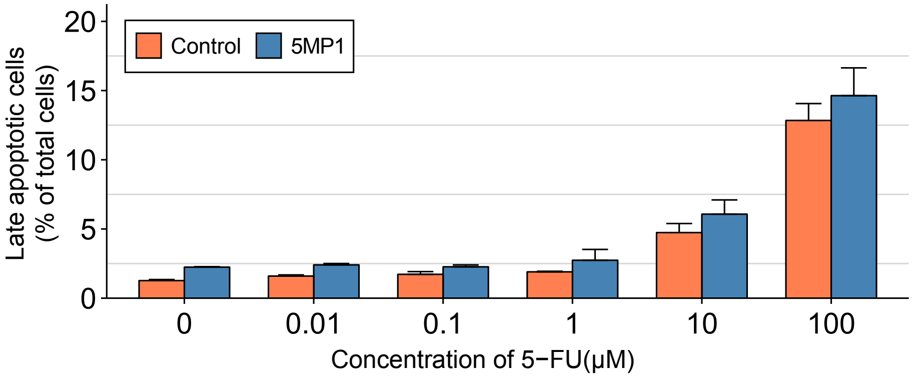

Figure S4. 5MP1 induces c-Myc-mediated cell cycle progression. Related to Figure 3. (a) Cell cycle assay of siRNA-mediated 5MP1-knockdown cells. Bar graphs represent the cell distribution in each cell cycle phase. (b) GSEA with a significant enrichment score showing a cell-cycle-related geneset that are positively correlated with *5MP1* expression in 5MP1-overexpressed cells. NES, normalized enrichment score. (c) Schematic of SCADS inhibitor screening analysis. (d) Representative data showing cell growth of control cells and 5MP1-overexpressed cells following treatment with each listed compound targeting the molecules displayed. (e) Apoptosis assay of 5MP1-overexpressed HCT116 cells. Bar graphs represent the cell distribution in late apoptosis (Annexin V-FITC+/propidium iodide [PI]+). Data represent the mean ± SD. (*) $p < 0.05$; (**) $p < 0.01$; (***) $p < 0.001$.
