# Supplementary material for: Novel oncogene 5MP1 reprograms c-Myc translation initiation to drive malignant phenotypes in colorectal cancer
Source: eBioMedicine. 2019 Jun 4;44:387–402. doi: 10.1016/j.ebiom.2019.05.058 (PMC6606960; doi:10.1016/j.ebiom.2019.05.058)
Supplement: Fig. S5 — Ribosome-protected profiles of c-Myc start codons in lactimidomycin (LTM)-treated HCT116 cells. Related to Figure 4. (a-c) The ribosome occupancy at CUG and AUG start codons of the MYC mRNA derived from LTM-treated HCT116 cells is shown as green peaks. All data of the indicated studies was obtained from the Trips-viz website (https://trips.ucc.ie/). [file mmc5.pdf]

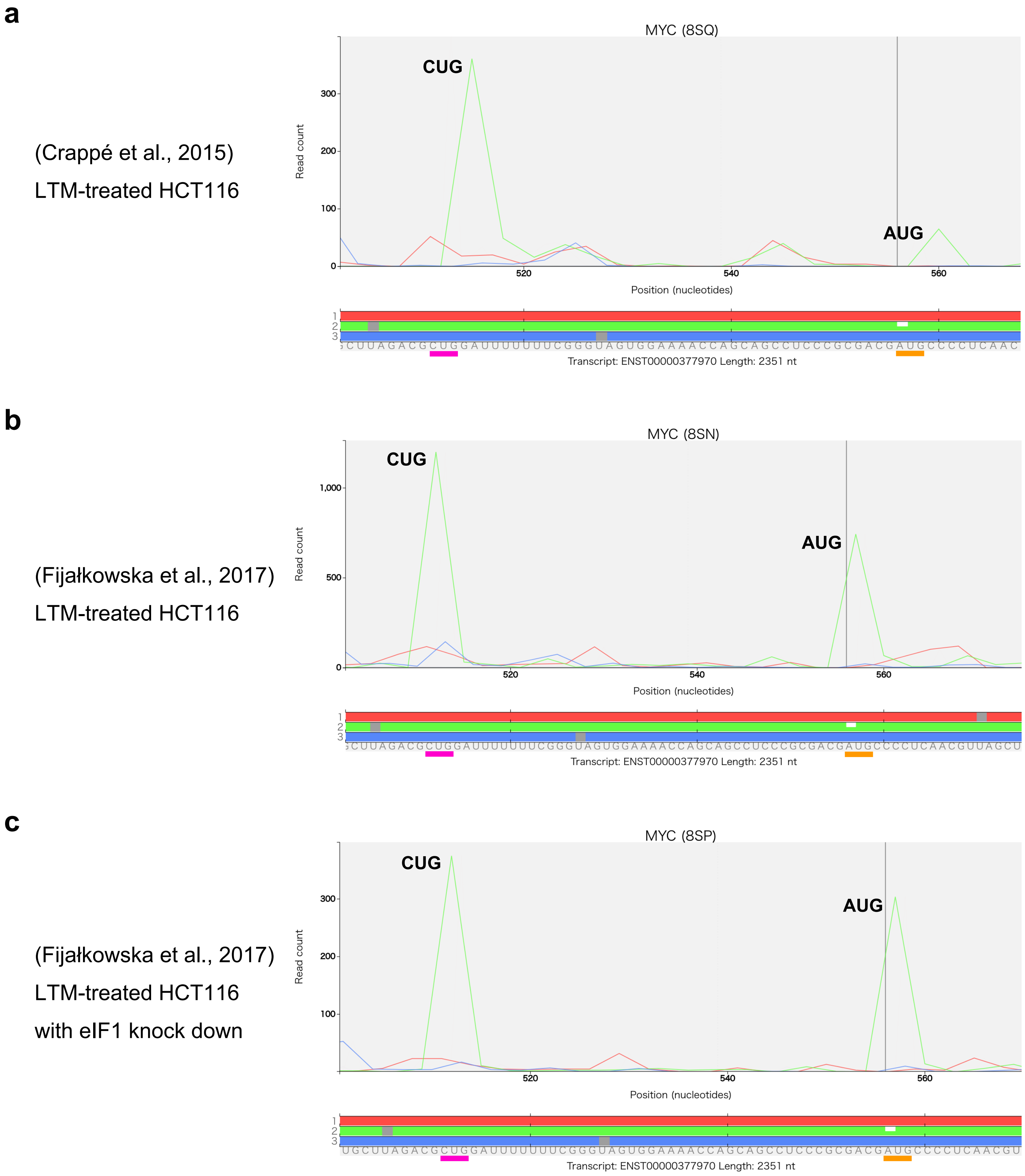

Figure S5. Ribosome-protected profiles of c-Myc start codons in lactimidomycin (LTM)-treated HCT116 cells. Related to Figure 4. (a-c) The ribosome occupancy at CUG and AUG start codons of the *MYC* mRNA derived from LTM-treated HCT116 cells is shown as green peaks. All data of the indicated studies was obtained from the Trips-viz website (https://trips.ucc.ie/).
