# Supplementary material for: Novel oncogene 5MP1 reprograms c-Myc translation initiation to drive malignant phenotypes in colorectal cancer
Source: eBioMedicine. 2019 Jun 4;44:387–402. doi: 10.1016/j.ebiom.2019.05.058 (PMC6606960; doi:10.1016/j.ebiom.2019.05.058)
Supplement: Fig. S6 — Transcriptional profiling of KMST-6 cells expressing AUG- and CUG-initiated c-Myc isoforms (isoform 1 and 2). Related to Figure5. (a) Transcriptomic distances of the KMST-6 stable cell lines expressing the indicated lentiviral vectors. (b) MA-plot of differentially expressed genes (DEGs) between the indicated KMST-6 stable cell lines. DEGs are represented as red dots. The cutoff of DEGs was determined as FDR < 0.01 (Likelihood ratio test). The numbers of significantly up- or downregulated genes are shown. (c) Hierarchical clustering of the top 50 upregulated genes (FDR < 0.01, likelihood ratio test) in isoform 1 (left) isoform 2 (right) compared to isoform 2 and isoform 1, respectively. Regularized log2 expression values are row-mean subtracted. (For interpretation of the references to color in this figure legend, the reader is referred to the web version of this article.) [file mmc6.pdf]

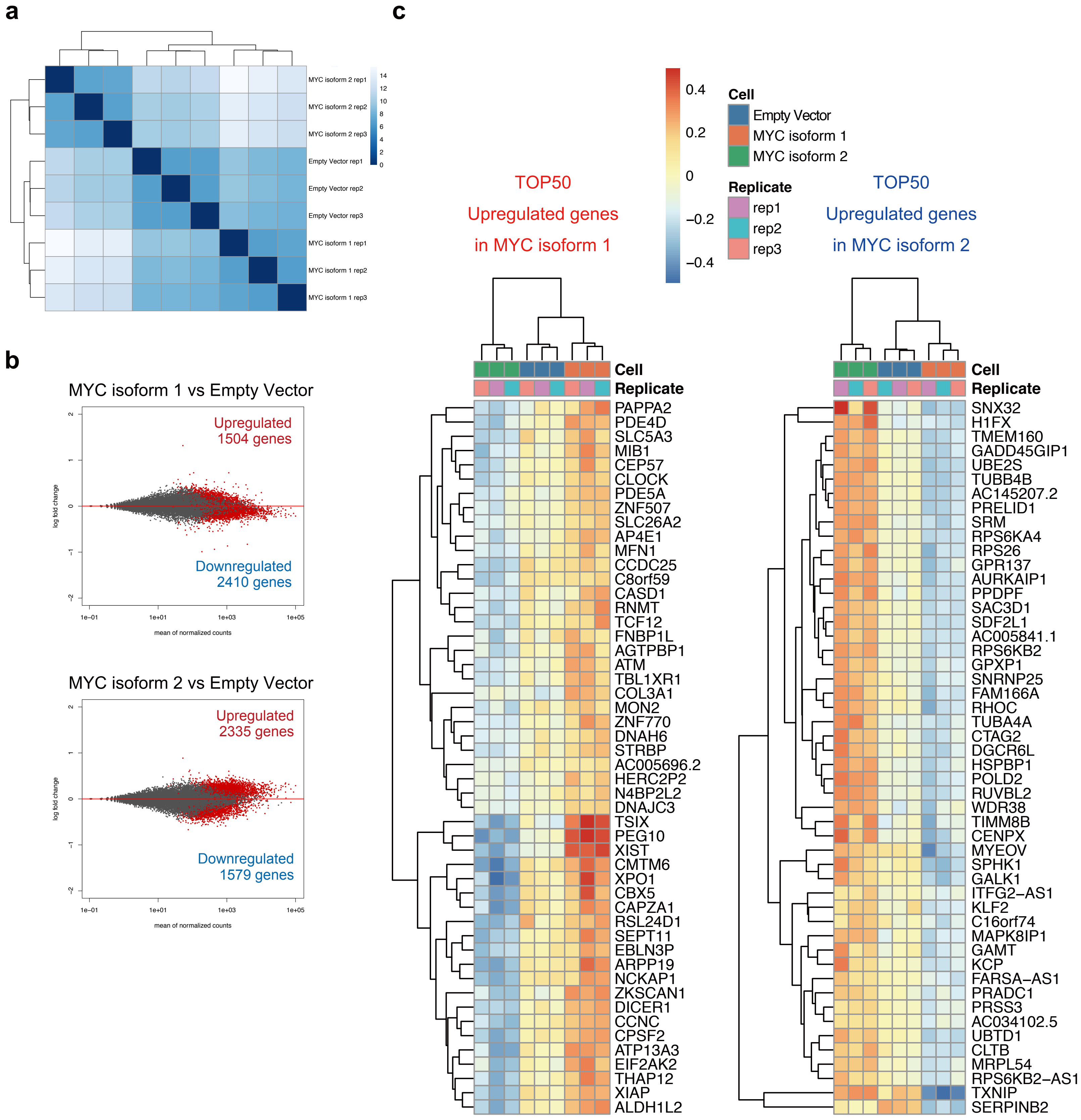

Figure S6. Transcriptional profiling of KMST-6 cells expressing AUG- and CUG-initiated c-Myc isoforms (isoform 1 and 2). Related to Figure 5. (a) Transcriptomic distances of the KMST-6 stable cell lines expressing the indicated lentiviral vectors. (b) MA-plot of differentially expressed genes (DEGs) between the indicated KMST-6 stable cell lines. DEGs are represented as red dots. The cutoff of DEGs was determined as FDR<0.01 (Likelihood ratio test). The numbers of significantly up- or downregulated genes are shown. (c) Hierarchical clustering of the top 50 upregulated genes (FDR < 0.01, likelihood ratio test) in isoform 1 (left) isoform 2 (right) compared to isoform 2 and isoform 1, respectively. Regularized log2 expression values are row-mean subtracted.
