# Supplementary material for: Novel oncogene 5MP1 reprograms c-Myc translation initiation to drive malignant phenotypes in colorectal cancer
Source: eBioMedicine. 2019 Jun 4;44:387–402. doi: 10.1016/j.ebiom.2019.05.058 (PMC6606960; doi:10.1016/j.ebiom.2019.05.058)
Supplement: Fig. S7 — Clinical relevance of 5MP2 and eIF5 expression levels in CRC. Related to Figure6. (a-b) Kaplan-Meier curves for the overall survival of CRC patients in the TCGA COADREAD dataset (N = 620), classified according to the 5MP2 mRNA expression levels (a) and eIF5 mRNA expression levels (b) in tumor tissues. P-values were calculated using the log-rank test. (c) eIF5 copy number in CRC tissues and non-neoplastic tissues of colorectal mucosa (NNT) in TCGA dataset. P represents p-values from the two-sided Mann-Whitney U test. (d) Violin plots of eIF5 mRNA expression levels in CRC tissues and NNT in the TCGA COADREAD dataset. P-values were calculated by pairwise comparisons using the Mann-Whitney U test with Bonferroni posttest. LG, Low Grade; HG, High Grade; n.s., not significant. (e) Representative images of immunohistochemical staining for eIF5 in CRC tissues (upper). Proportions of eIF5 levels in tumor tissues and NNT are shown using three-stage staining score (lower). T, Tumor; N, NNT; scale bars, 200 μm. (f) Violin plots of eIF5 protein expression levels in CRC tissues and NNT in the TCGA COADREAD dataset. P represents p-values from the two-sided Mann-Whitney U test. [file mmc7.pdf]

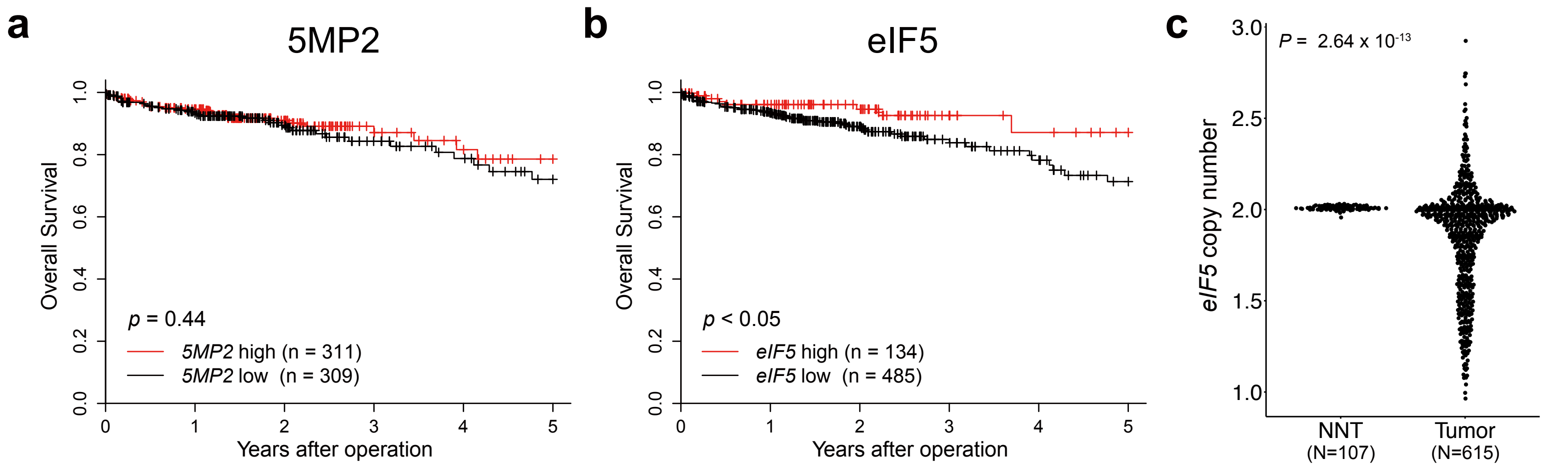

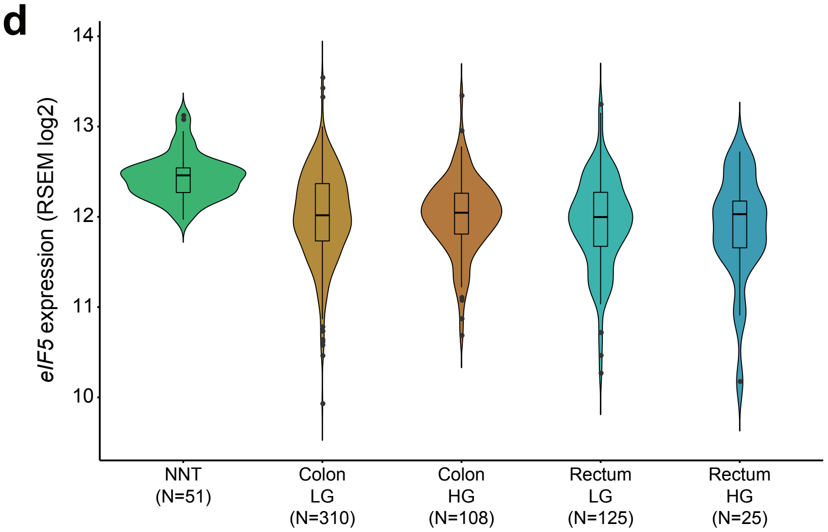

Comparison	P-Value
NNT vs Colon LG	2.0×10^{-9}
NNT vs Colon HG	2.1×10^{-10}
NNT vs Rectum LG	6.1×10^{-5}
NNT vs Rectum HG	1.2×10^{-10}
Colon LG vs Colon HG	n.s.
Colon LG vs Rectum LG	n.s.
Colon LG vs Rectum HG	n.s.
Colon HG vs Rectum LG	n.s.
Colon HG vs Rectum HG	n.s.
Rectum LG vs Rectum HG	n.s.

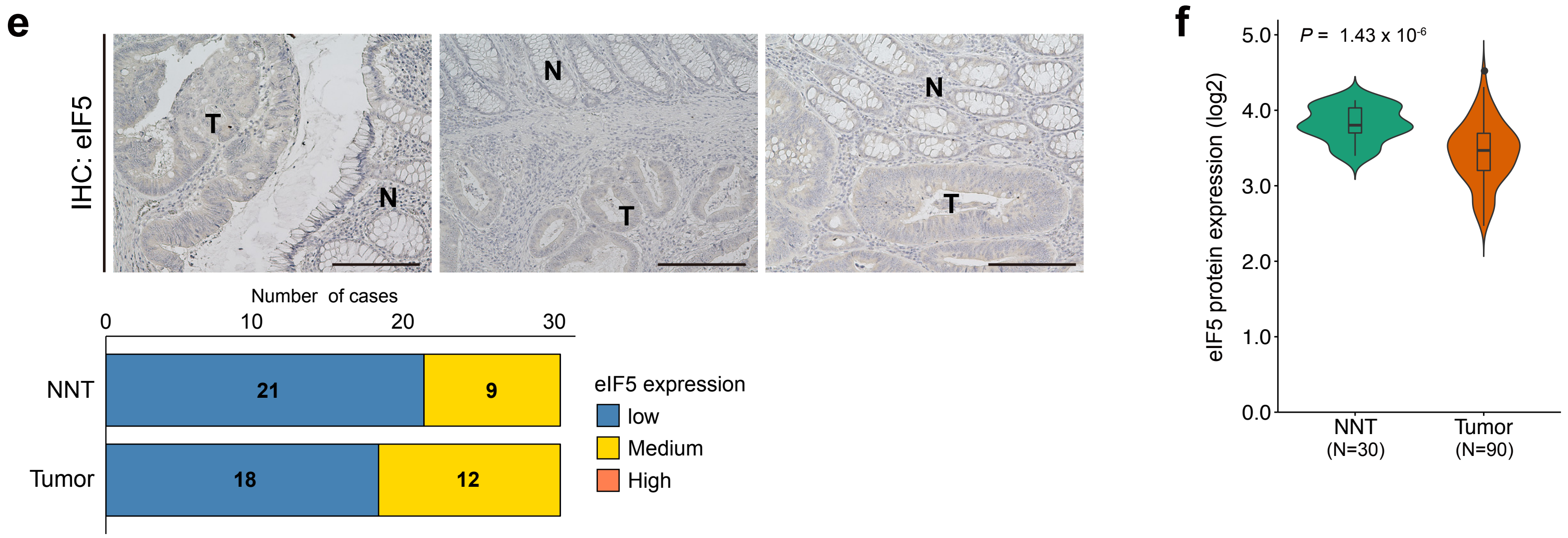

Figure S7. Clinical relevance of 5MP2 and eIF5 expression levels in CRC. Related to Figure 6.
(a-b) Kaplan-Meier curves for the overall survival of CRC patients in the TCGA COADREAD dataset (N = 620), classified according to the *5MP2* mRNA expression levels (a) and *eIF5* mRNA expression levels (b) in tumor tissues. P-values were calculated using the log-rank test. (c) *eIF5* copy number in CRC tissues and non-neoplastic tissues of colorectal mucosa (NNT) in TCGA dataset. P represents p-values from the two-sided Mann-Whitney U test. (d) Violin plots of *eIF5* mRNA expression levels in CRC tissues and NNT in the TCGA COADREAD dataset. P-values were calculated by pairwise comparisons using the Mann-Whitney U test with Bonferroni posttest. LG, Low Grade; HG, High Grade; n.s., not significant. (e) Representative images of immunohistochemical staining for eIF5 in CRC tissues (upper). Proportions of eIF5 levels in tumor tissues and NNT are shown using three-stage staining score (lower). T, Tumor; N, NNT; scale bars, 200μm. (f) Violin plots of eIF5 protein expression levels in CRC tissues and NNT in the TCGA COADREAD dataset. *P* represents p-values from the two-sided Mann-Whitney U test.
