# Supplementary material for: Novel oncogene 5MP1 reprograms c-Myc translation initiation to drive malignant phenotypes in colorectal cancer
Source: eBioMedicine. 2019 Jun 4;44:387–402. doi: 10.1016/j.ebiom.2019.05.058 (PMC6606960; doi:10.1016/j.ebiom.2019.05.058)
Supplement: Table S1 — Resources used in this study. [file mmc8.docx]

**Table S1: Resources used in this study.**

| **Reagent or Resource** | **Supplier or Reference** | **Identifier or Web Link** |
| --- | --- | --- |
| **Antibodies** | | |
| Rabbit polyclonal anti-5MP1 | Abcam | Cat# ab96682, RRID: AB_10678870 |
| Rabbit polyclonal anti-5MP1 | GeneTex | Cat# GTX106985, RRID: AB_1949773 |
| Rabbit polyclonal anti-eIF5 | GeneTex | Cat# GTX114923, RRID: AB_10730759 |
| Rabbit monoclonal anti-c-Myc | Abcam | Cat# ab32072, RRID: AB_731658 |
| Rabbit monoclonal anti-CDK2 | Abcam | Cat# ab32147, RRID: AB_726775 |
| Rabbit polyclonal anti-Ki67 | Abcam | Cat# ab15580, RRID: AB_443209 |
| Mouse monoclonal anti-b-actin | Santa Cruz | Cat# sc-47778, RRID: AB_626632 |
| Cell Cycle (pCDK/pHH3/Actin) WB Cocktail | Abcam | Cat# ab136810 |
| Rabbit monoclonal secondary antibody | GE Healthcare | Cat# NA934, RRID: AB_772206 |
| Mouse monoclonal secondary antibody | GE Healthcare | Cat# NA931, RRID: AB_772210 |
| **Bacterial and Virus Strains** | | |
| E. coli JM109 Competent Cells | Takara | Cat# 9052 |
| One Shot TOP10 Chemically Competent E. coli | Thermo Fisher | Cat# C404010 |
| **Chemicals, Peptides, and Recombinant Proteins** | | |
| Lipofectamine® 3000 Transfection Reagent | Thermo Fisher | Cat# L3000008 |
| Lipofectamine® RNAiMAX Transfection Reagent | Thermo Fisher | Cat# 13778075 |
| Puromycin | Thermo Fisher | Cat# A1113803 |
| Cycloheximide | Abcam | Cat# ab120093 |
| 5-fluorouracil | Wako | Cat# 066-01402 |
| B27 Supplement | Thermo Fisher | Cat# 17504044 |
| Recombinant Human EGF | PeproTech | Cat# AF-100-15 |
| Recombinant Human FGF-basic | PeproTech | Cat# 100-18B |
| Propidium Iodide | Sigma Aldrich | Cat# P4170 |
| RNAlater™ Stabilization Solution | Thermo Fisher | Cat# AM7020 |
| PhosStop™ phosphatase inhibitor | Roche | Cat# 4906845001 |
| Protease Inhibitor Cocktail | BioVision | Cat# K271 |
| Phusion High-Fidelity DNA Polymerase | New England Biolabs | Cat# M0530 |
| M-MLV reverse transcriptase | Thermo Fisher | Cat# 28025013 |
| qPCR Human Reference Total RNA | Clontech | Cat# 636690 |
| **Critical Commercial Assays** | | |
| Annexin V-FITC Apoptosis Detection Kit | Abcam | Cat# ab14085 |
| BCA Protein Assay Kit | Thermo Fisher | Cat# 23225 |
| LightCycler® 480 SYBR Green I Master | Roche | Cat# 04887352001 |
| ISOGEN-II | Nippon Gene | Cat# 311-07361 |
| KOD-Plus-Mutagenesis Kit | Toyobo Life Science | Cat# SMK-101 |
| REAL Envision™ Kit | Dako | Cat# K5007 |
| Differential Quick Stain Kit | Sysmex | Cat# 16920 |
| Cell Proliferation Kit I | Roche | Cat# 11465007001 |
| Dual-Glo® Luciferase Assay System | Promega | Cat# E2920 |
| **Deposited Data** | | |
| Human: TCGA-COADREAD dataset (RSEM normalized gene expression, GISTIC2 Copy number and clinical data) | The Cancer Genome Atlas Network | https://gdac.broadinstitute.org |
| Human: TCGA-COADREAD dataset (somatic mutation and clinical data) | NIH Genomic Data Commons Legacy Archive | https://portal.gdc.cancer.gov/legacy-archive |
| Human: Mass spectrometry dataset of colorectal cancer | [3] | https://cptac-data-portal.georgetown.edu/cptac/s/S022 |
| Human: Microarray dataset of colorectal cancer | [50] | GEO: GSE21815 |
| Human: Microarray dataset of colorectal cancer | [49] | GEO: GSE32323 |
| Human: Microarray dataset of colorectal adenoma | [52] | GEO: GSE8671 |
| Human: RNA-seq dataset of 5MP1-overexpressed HCT116 cells | This Paper | GEO: GSE118105 |
| Human: RNA-seq dataset of c-Myc-overexpressed KMST6 cells | This Paper | GEO: GSE118113 |
| **Experimental Models: Cell Lines** | | |
| HCT116 | RIKEN BRC | Cat# RCB2979 |
| SW480 | ATCC | Cat# CCL-228 |
| LoVo | JCRB | Cat# JCRB9083 |
| RCM1 | JCRB | Cat# JCRB0256 |
| HEK293T | RIKEN BRC | Cat# RCB2202 |
| KMST-6 | RIKEN BRC | Cat# RCB1955 |
| **Experimental Models: Organisms/Strains** | | |
| Mouse: BALB/cSlc-*nu/nu* | Japan SLC | N/A |
| **Oligonucleotides** | | |
| Control siRNA-A (Negative Control) | Santa Cruz | Cat# sc-37007 |
| Silencer Select siRNA targeting *5MP1* #1 | Applied Biosystems | Cat# s26319 |
| Silencer Select siRNA targeting *5MP1* #2 | Applied Biosystems | Cat# s26320 |
| qPCR primers: *5MP1* (Forward: 5'-CATTGCGGCCTCATTTGCTG-3'; Reverse: 5'-CGGAGGAAGTCGGAAAGCTC-3') | This paper | N/A |
| qPCR primers: *MYC* (Forward: 5'- CATCAGCACAACTACGCAGC-3'; Reverse: 5'- GCTGGTGCATTTTCGGTTGT-3') | This paper | N/A |
| qPCR primers: *CDC25A* (Forward: 5'- AGAATGGGCTCCTCCGAGTC-3'; Reverse: 5'- CTGGACTACATCCCAACAGCTT-3') | This paper | N/A |
| qPCR primers: *CDK4* (Forward: 5'- CTCTCTAGCTTGCGGCCT-3'; Reverse: 5'- ATCGAGAGGTAGCCATTCTCAG-3') | This paper | N/A |
| qPCR primers: *CCNE1* (Forward: 5'- GTTTACCCAAACTCAACGTGC-3'; Reverse: 5'- CGCAAACTGGTGCAACTTTGG-3') | This paper | N/A |
| qPCR primers: *GAPDH* (Forward: 5'- AGCCACATCGCTCAGACAC-3'; Reverse: 5'- GCCCAATACGACCAAATCC-3') | This paper | N/A |
| qPCR primers: *RPS18* (Forward: 5'-AGTCCCTGCCCTTTGTACACA -3'; Reverse: 5'-CGATCCGAGGGCCTCACTA -3') | This paper | N/A |
| Cloning primers: *5MP1* (Forward: BamHI-*5MP1*; 5'- ggatccACAGGCCAGCGGTTCAAAAC -3'; Reverse: EcoRI-*5MP1*; 5'- gaattcCCTTTGCGAAAACAGAAGCCAAG -3') | This paper | N/A |
| Cloning primers: *MYC (AUG)* (Forward: BamHI-*MYC*; 5'- ggatccAGTGGAAAACCAGCAGCCTCC -3'; Reverse: EcoRI-*MYC*; 5'- gaattcCTCAAGACTCAGCCAAGGTTGT -3') | This paper | N/A |
| Cloning primers: *MYC (CUG)* (Forward: BamHI-*MYC*; 5'- ggatccCCGCTTCTCTGAAAGGCTCTCC -3'; Reverse: EcoRI-*MYC*; 5'- gaattcGCCAAGGTTGTGAGGTTGCAT -3') | This paper | N/A |
| Site-directed Mutagenesis primers: *CTG* to *ATG* in the *MYC (CUG)* plasmid (Forward: 5'- ATGGATTTTTTTCGGGTAGTGGAAA -3'; Reverse: 5'- CGTCTAAGCAGCTGCAAGGAGAG -3') | This paper | N/A |
| Site-directed Mutagenesis primers: *ATG* to *AAG* in the *MYC (CUG)* plasmid (Forward: 5'- AAGCCCCTCAACGTTAGCTTCACCA -3'; Reverse: 5'- CGTCGCGGGAGGCTGCTGGTTTTCC -3') | This paper | N/A |
| **Recombinant DNA** | | |
| pCDF1-MCS2-EF1-Puro | System Biosciences | Cat# CD110B-1 |
| pCDF1-5MP1-EF1-Puro | This paper | N/A |
| pCDF1-MYC(AUG)-EF1-Puro | This paper | N/A |
| pCDF1-MYC(CUG)-EF1-Puro | This paper | N/A |
| c-Myc_408 Firefly Luciferase WT plasmid | [31] | N/A |
| c-Myc_408 Firefly Luciferase Mutant plasmid | [31] | N/A |
| **Software and Algorithms** | | |
| Genomon2 v2.5.0 | Human Genome Center (The University of Tokyo) | https://genomon-project.github.io/GenomonPagesR |
| STAR v2.5.2a | [38] | https://github.com/alexdobin/STAR |
| HTSeq v0.6.0 | [39] | https://htseq.readthedocs.io/ |
| R v3.2.3 | The R Foundation | https://www.r-project.org |
| R Studio v1.1.383 | R Studio | https://www.rstudio.com |
| BioConductor v3.2 | [40] | http://www.bioconductor.org |
| DESeq2 v1.10.1 | [41] | http://www.bioconductor.org/packages/ release/bioc/html/DESeq2.html |
| dNdScv v0.0.1.0 | [37] | https://github.com/im3sanger/dndscv |
| GSEA | [43] | http://software.broadinstitute.org/gsea/index.jsp |
| DAVID | [42] | https://david.ncifcrf.gov/ |
| LinkedOmics | [36] | http://www.linkedomics.org |
| ImageJ v1.50c4 | National Institutes of Health | https://imagej.nih.gov/ij |
| IHC Profiler | [34] | https://sourceforge.net/projects/ihcprofiler |
| JMP Pro v13.0.0 | SAS Institute | https://www.jmp.com/en_us/home.html |
| **Other** | | |
| SCADS inhibitor kit | The Screening Committee of Anticancer Drugs | http://scads.jfcr.or.jp/kit/kit.html |
